# Supplementary figures and images for: Distinctive types of postzygotic single-nucleotide mosaicisms in healthy individuals revealed by genome-wide profiling of multiple organs
Source: PLoS Genet. 2018 May 15;14(5):e1007395. doi: 10.1371/journal.pgen.1007395 (PMC5969758; doi:10.1371/journal.pgen.1007395)

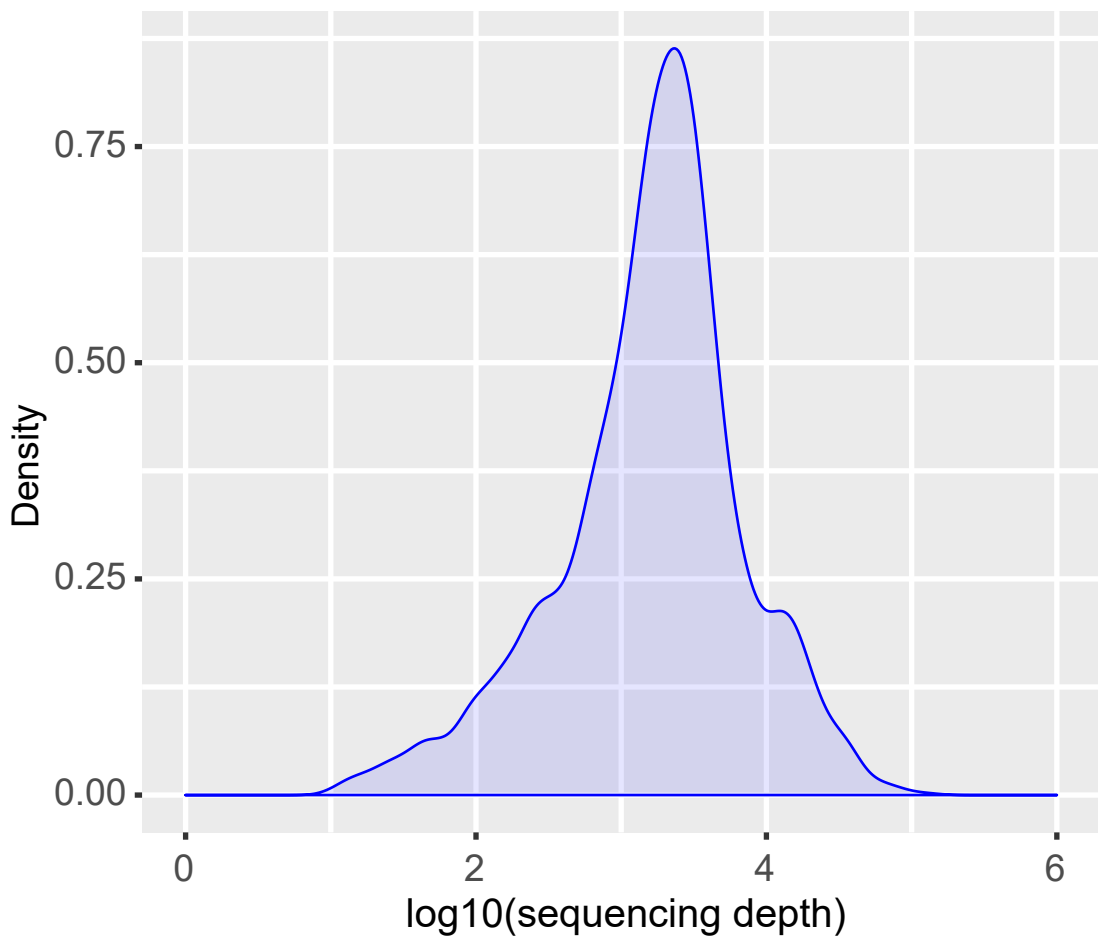

Supplement: S1 Fig — The average depth was greater than 4000X and more than 90% of the candidate sites were sequenced with at least 200X coverage in each sample. (PDF) [file pgen.1007395.s001.pdf]

BBL1100C

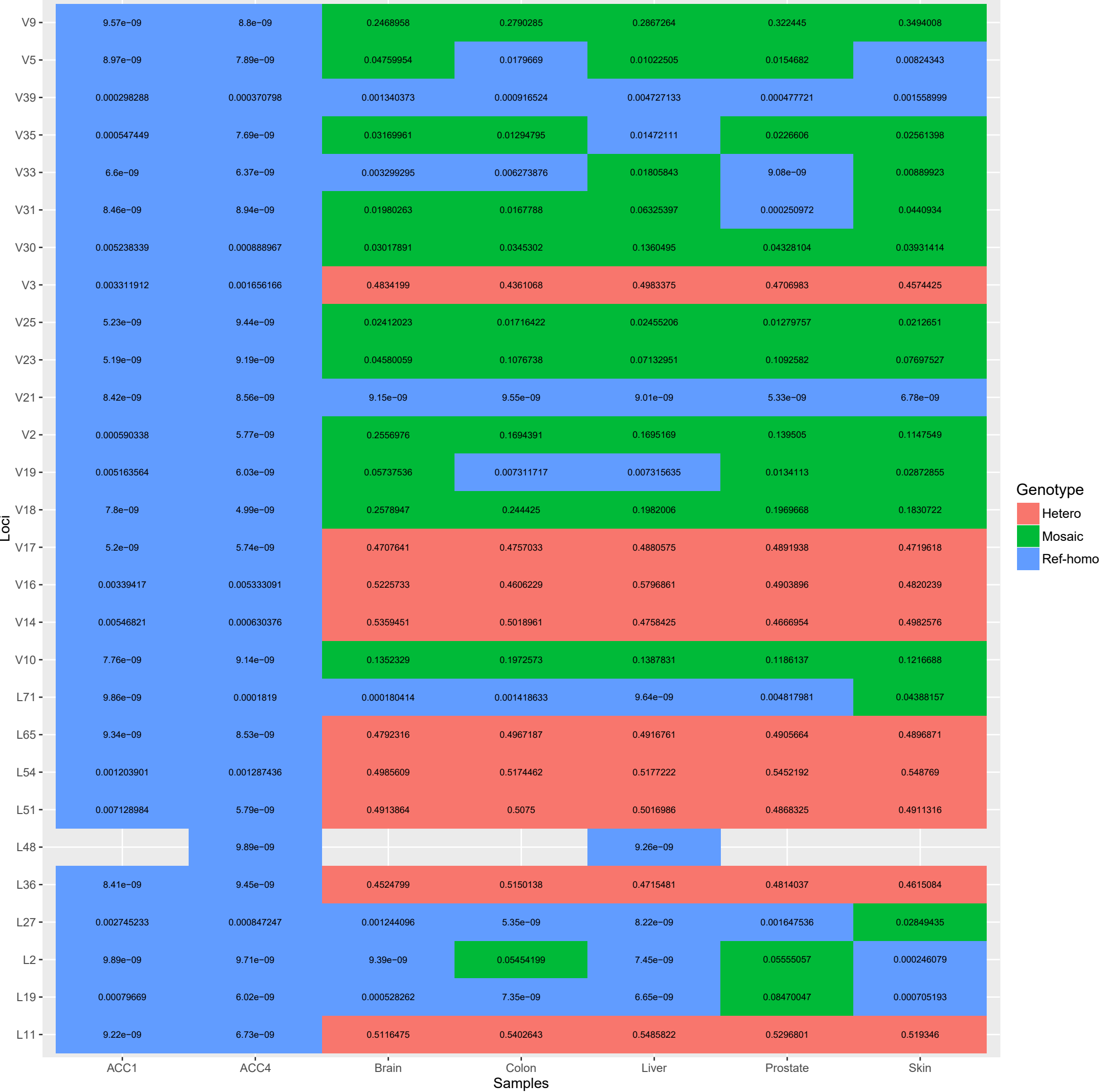

Supplement: S2 Fig — ACC1 and ACC4 are two unrelated individuals that served as negative controls. The mutant allele fractions were assessed by PGM Amplicon Sequencing of Mosaicism (PASM). Red, green, and blue colors denote heterozygous, mosaic, and reference-homozygous genotypes, respectively. (PDF) [file pgen.1007395.s002.pdf]

## BBLC1013

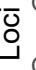

Supplement: S4 Fig — ACC1 and ACC4 are two unrelated individuals that served as negative controls. The mutant allele fractions were assessed by PGM Amplicon Sequencing of Mosaicism (PASM). Red, green, and blue colors denote heterozygous, mosaic, and reference-homozygous genotypes, respectively. (PDF) [file pgen.1007395.s004.pdf]

BBLD1005

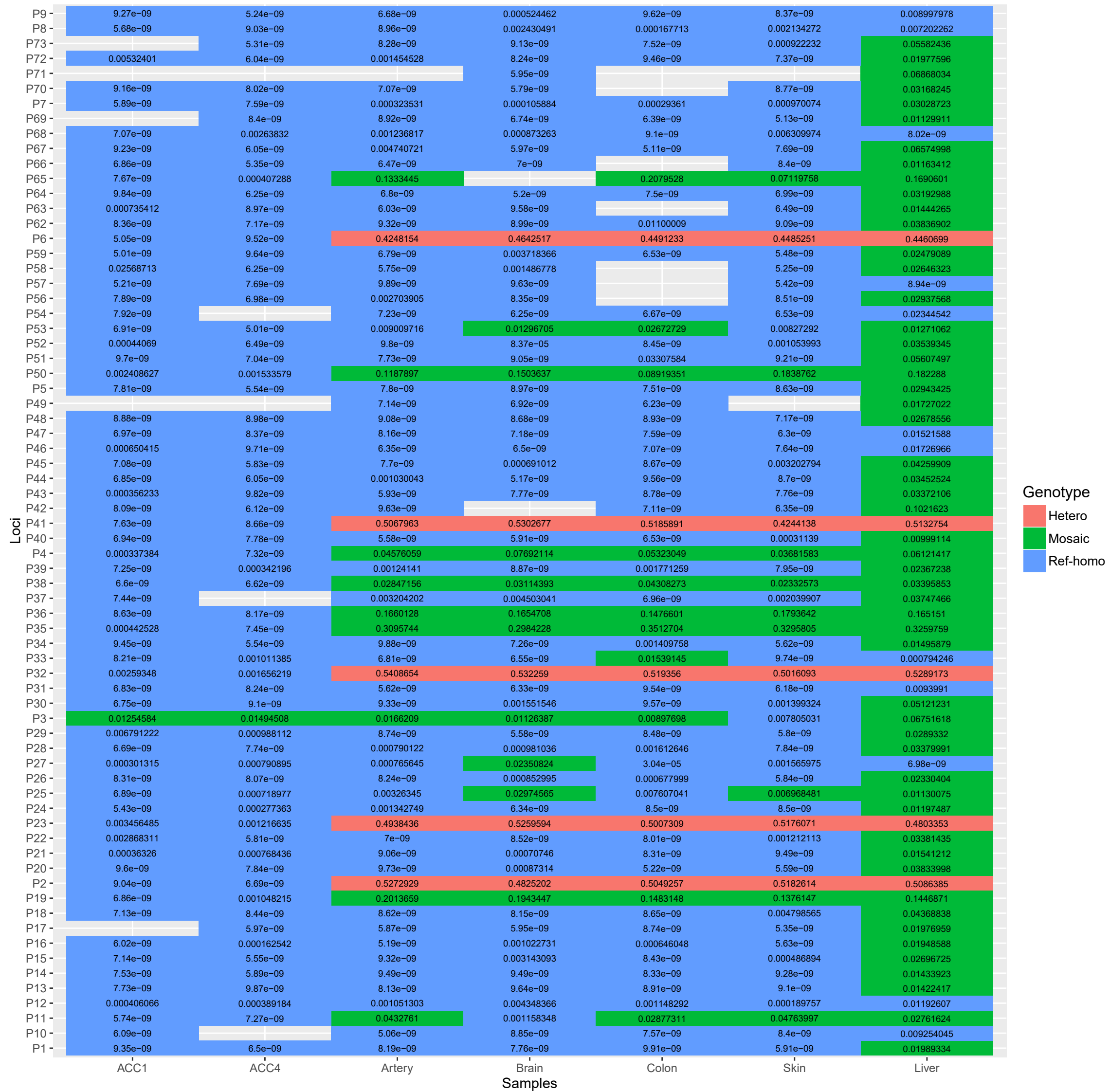

Supplement: S5 Fig — ACC1 and ACC4 are two unrelated individuals that served as negative controls. The mutant allele fractions were assessed by PGM Amplicon Sequencing of Mosaicism (PASM). Red, green, and blue colors denote heterozygous, mosaic, and reference-homozygous genotypes, respectively. (PDF) [file pgen.1007395.s005.pdf]

BBLD1010

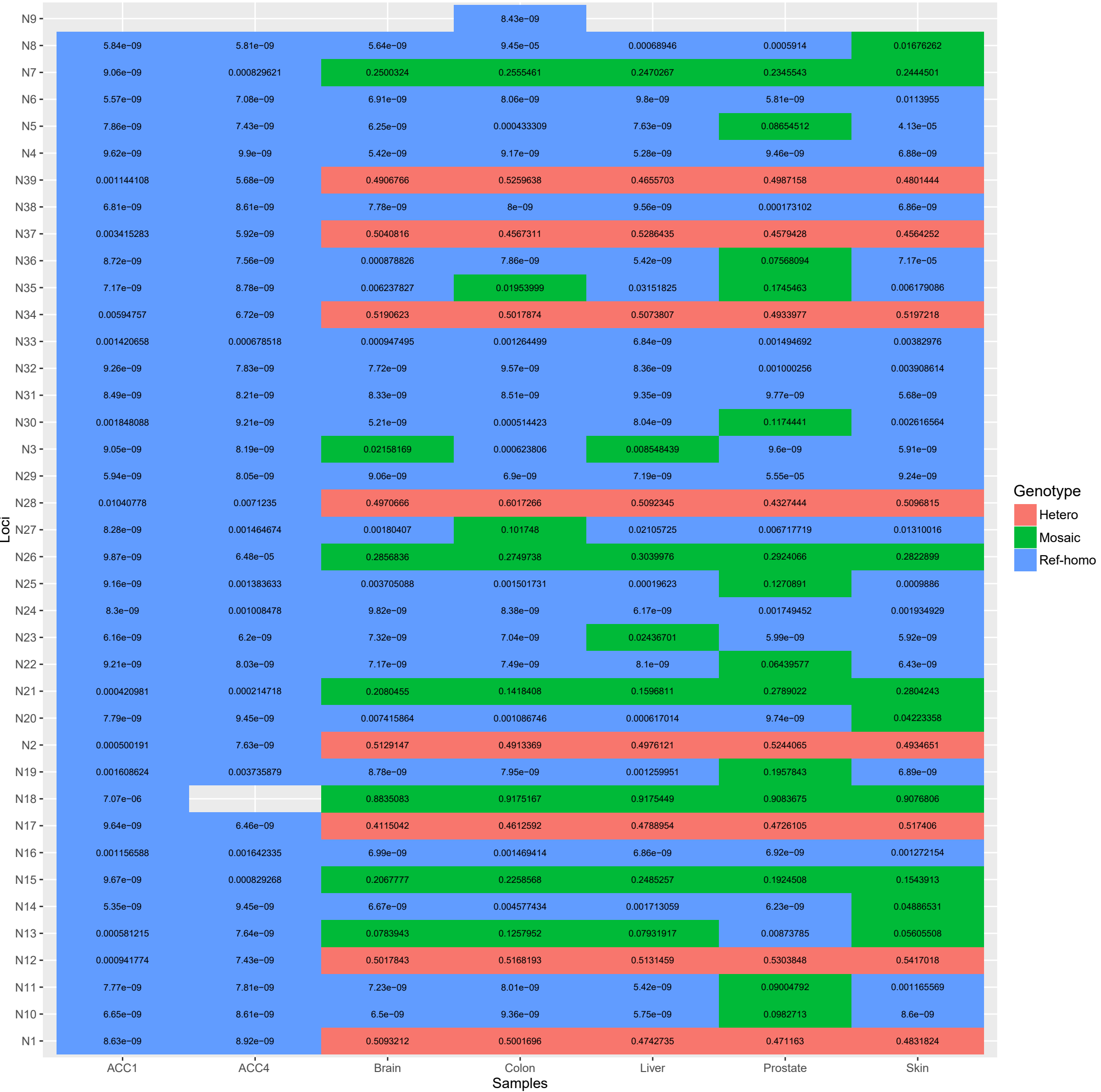

Supplement: S6 Fig — ACC1 and ACC4 are two unrelated individuals that served as negative controls. The mutant allele fractions were assessed by PGM Amplicon Sequencing of Mosaicism (PASM). Red, green, and blue colors denote heterozygous, mosaic, and reference-homozygous genotypes, respectively. (PDF) [file pgen.1007395.s006.pdf]

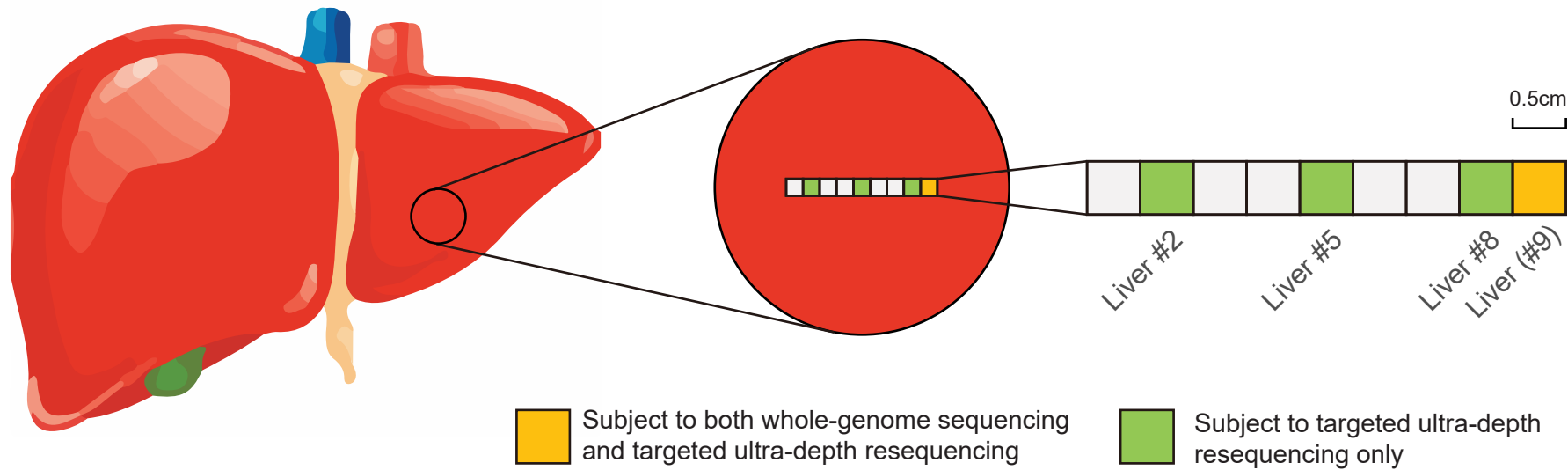

Supplement: S7 Fig — Targeted ultra-deep resequencing was performed on three additional liver samples (liver #2, #5, and #8) with varied physical distances to the original whole-genome sequenced liver sample (liver #9). (PDF) [file pgen.1007395.s007.pdf]

BBLD1005

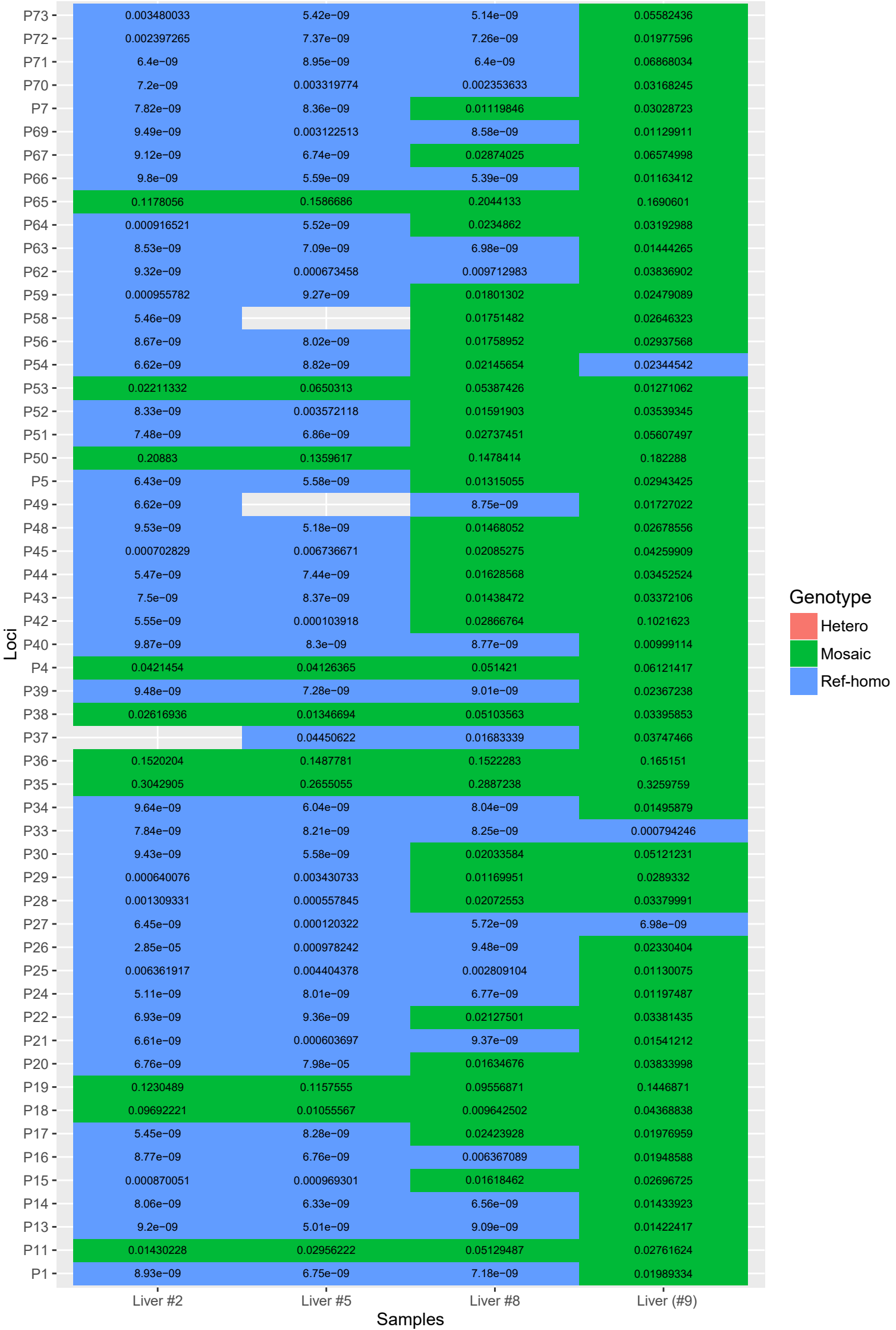

Supplement: S8 Fig — The mutant allele fractions were assessed by PGM Amplicon Sequencing of Mosaicism (PASM). Red, green, and blue colors denote heterozygous, mosaic, and reference-homozygous genotypes, respectively. (PDF) [file pgen.1007395.s008.pdf]

BBL11121

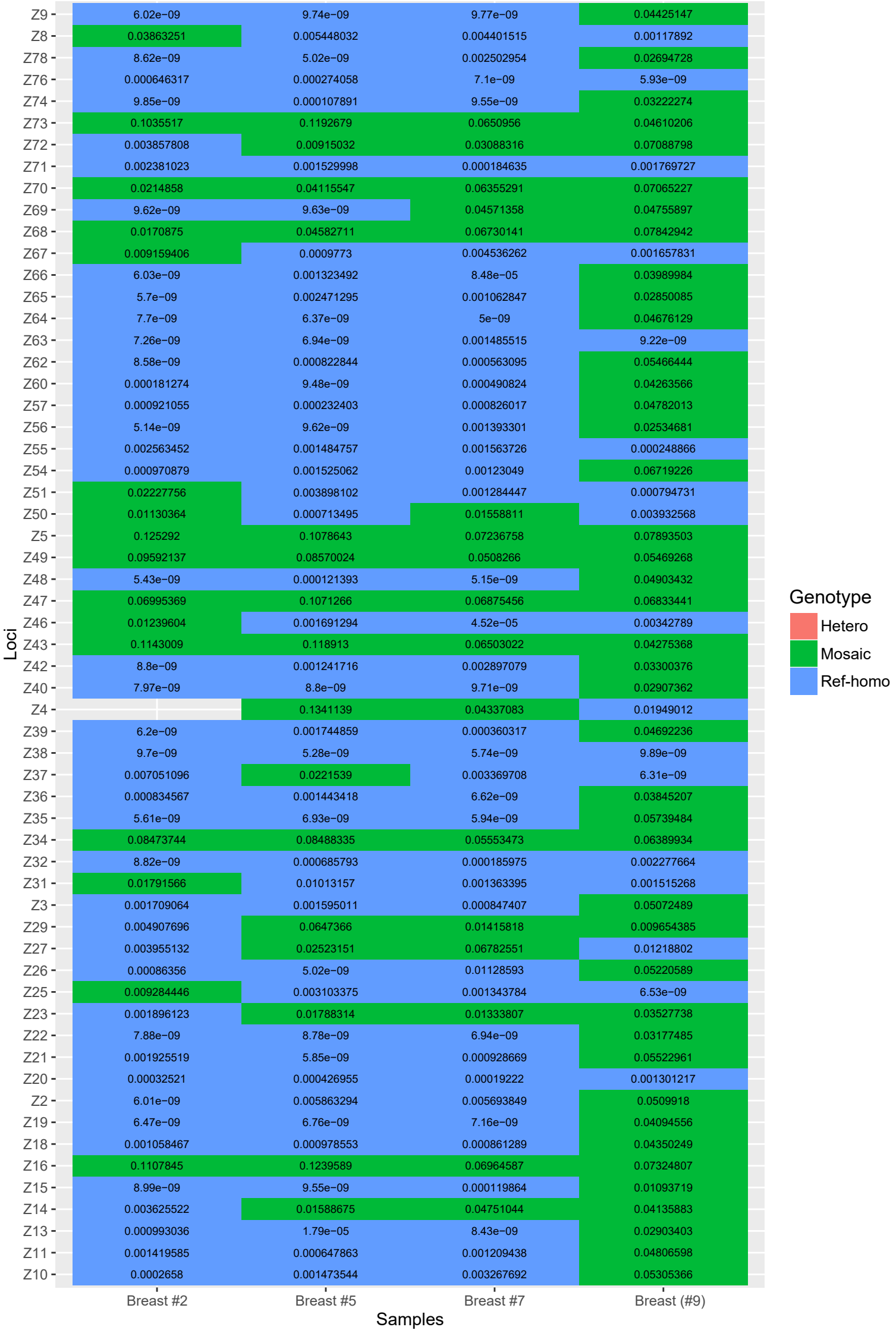

Supplement: S9 Fig — The mutant allele fractions were assessed by PGM Amplicon Sequencing of Mosaicism (PASM). Red, green, and blue colors denote heterozygous, mosaic, and reference-homozygous genotypes, respectively. (PDF) [file pgen.1007395.s009.pdf]

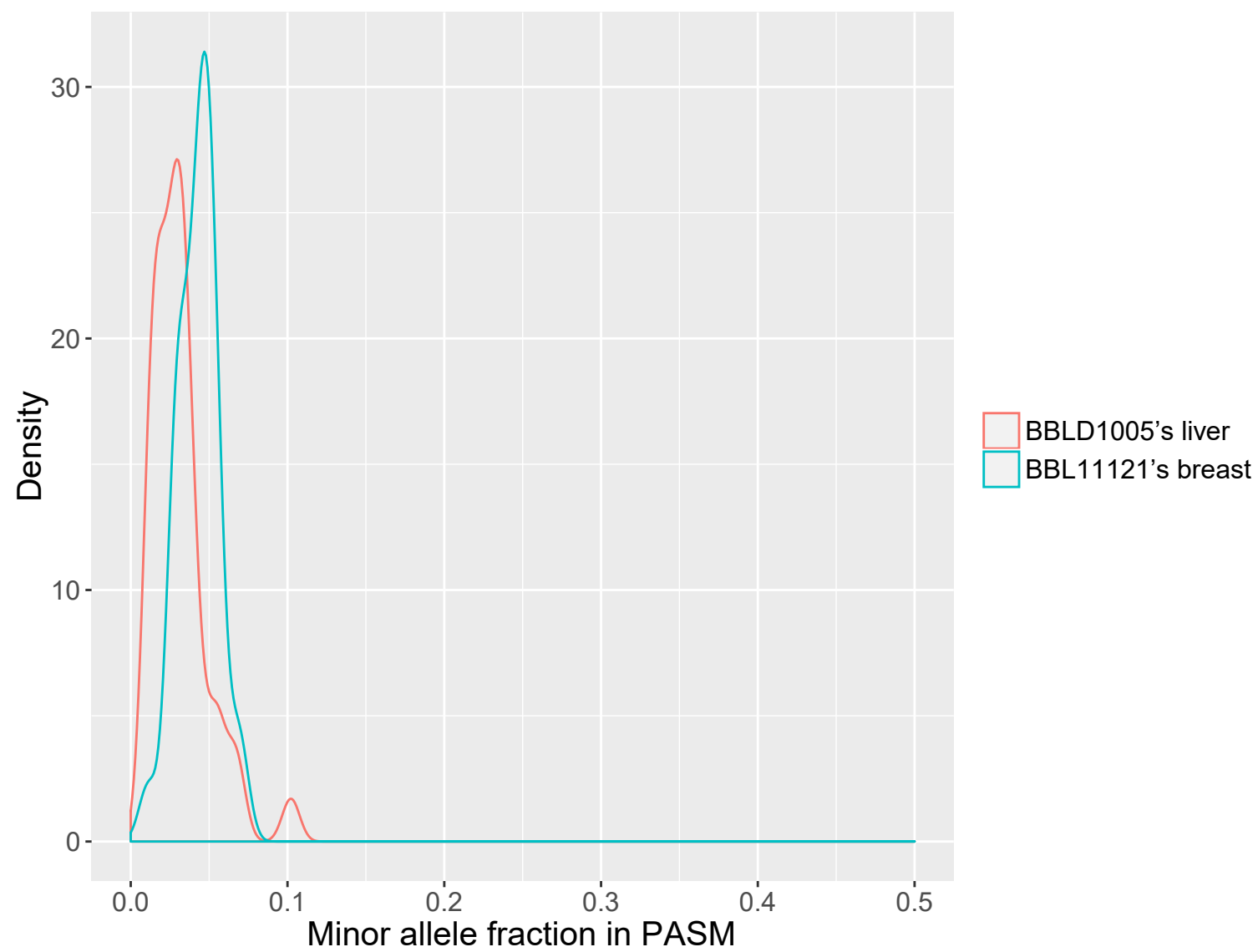

Supplement: S10 Fig — Each sample showed a single peak for the mosaic allele fraction, suggesting that they originated from clonal expansion. (PDF) [file pgen.1007395.s010.pdf]

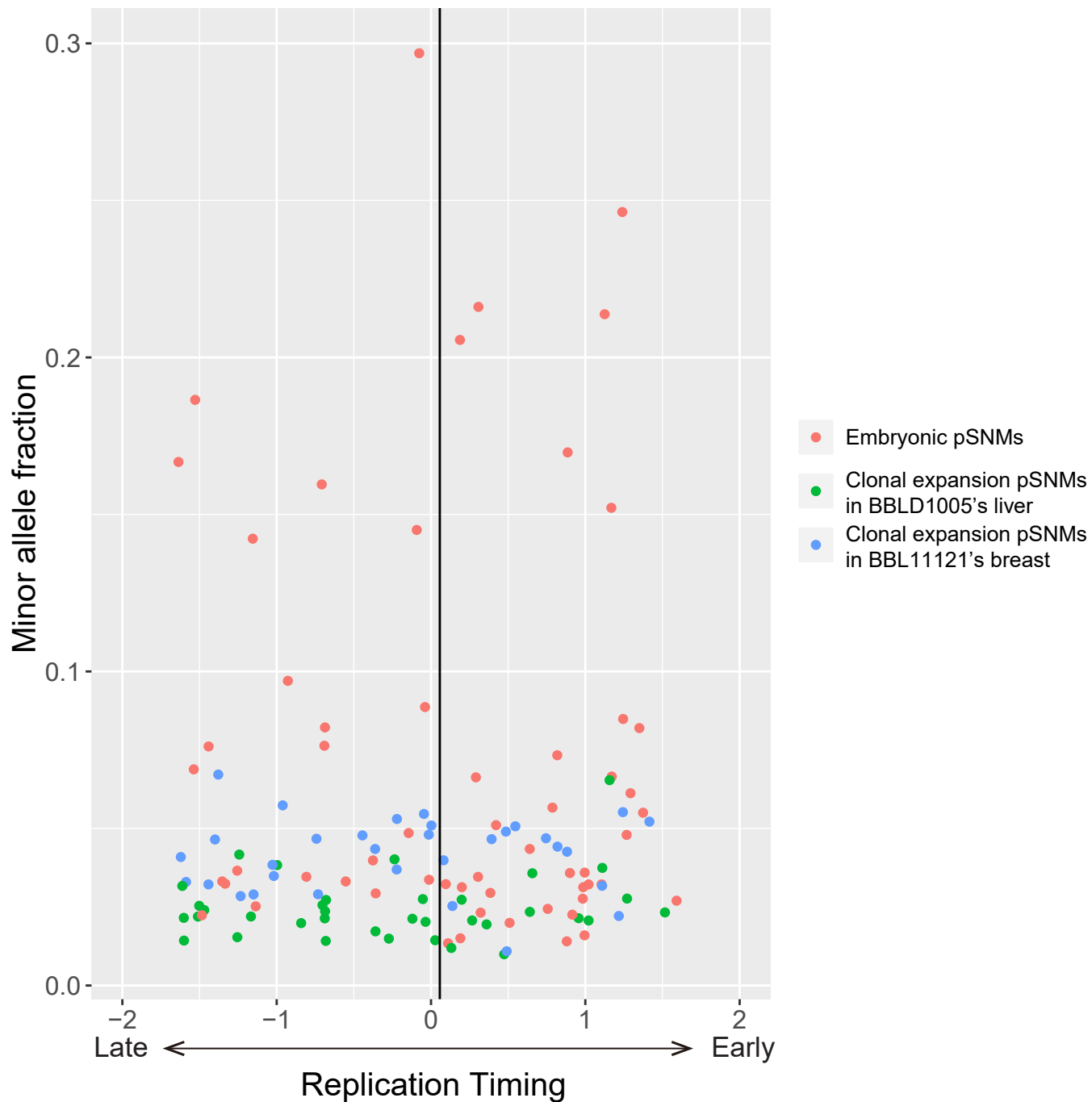

Supplement: S11 Fig — The grey line denotes the genomic average. Embryonic pSNMs with a wide range of allele fractions contributed to the enrichment of early-replicating regions. (PDF) [file pgen.1007395.s011.pdf]

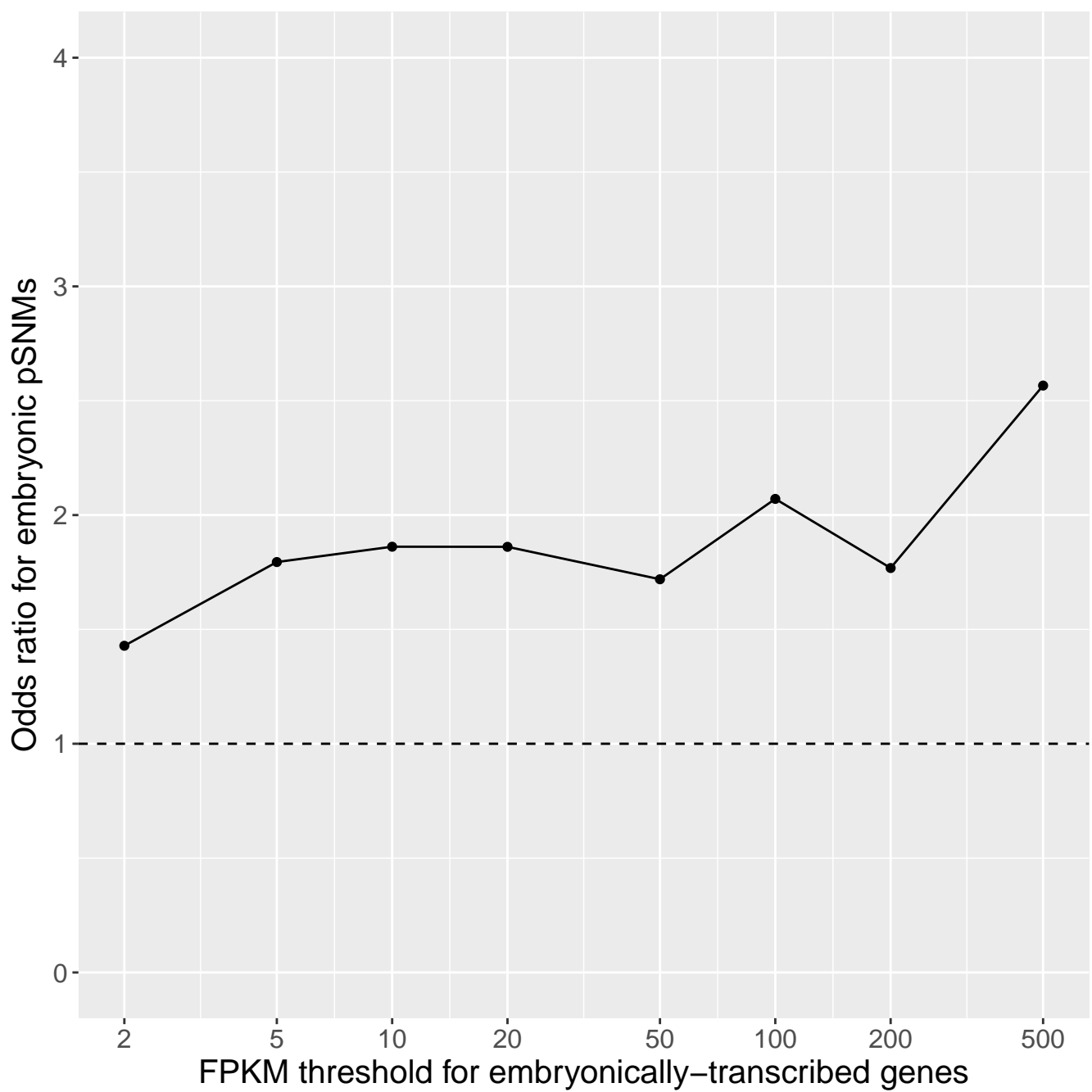

Supplement: S12 Fig — The X axis denotes different FPKM thresholds to define embryonically-transcribed genes, and the Y axis denotes the odds ratio of enrichment between embryonic and non-embryonic pSNMs. The odds ratios were robustly greater than one with varied FPKM thresholds. (PDF) [file pgen.1007395.s012.pdf]

HepG2

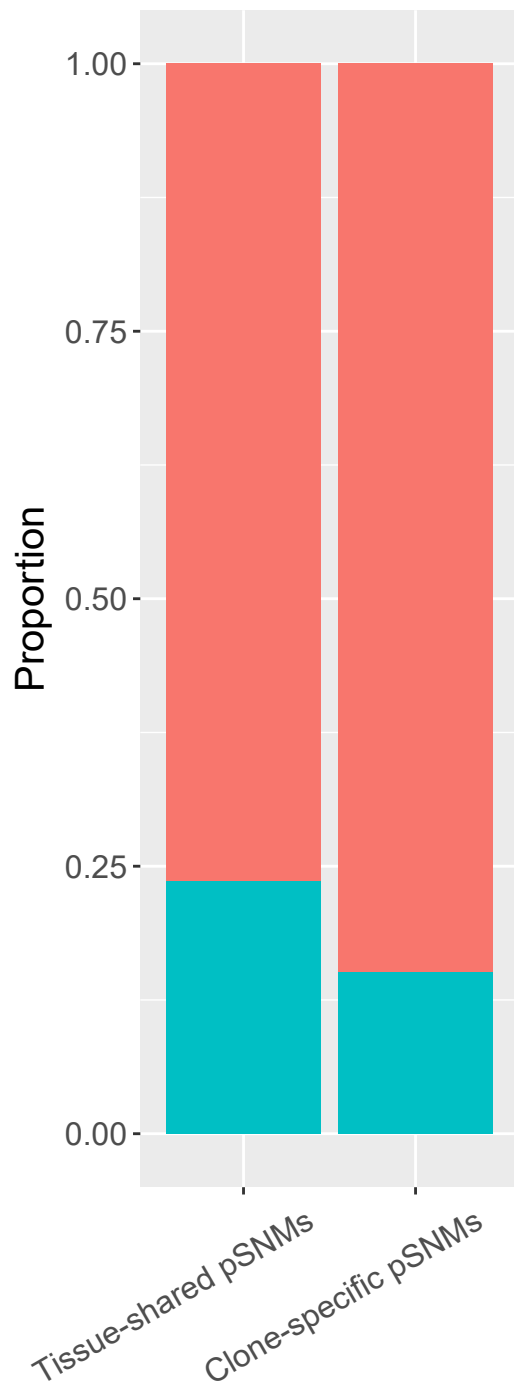

HMEC

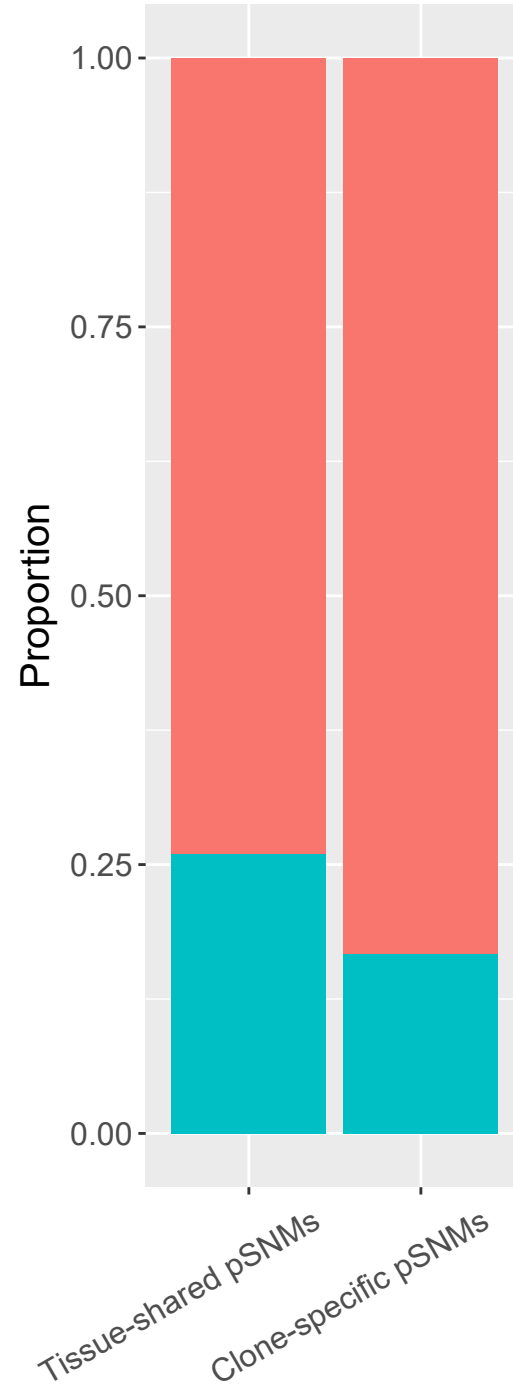

K562

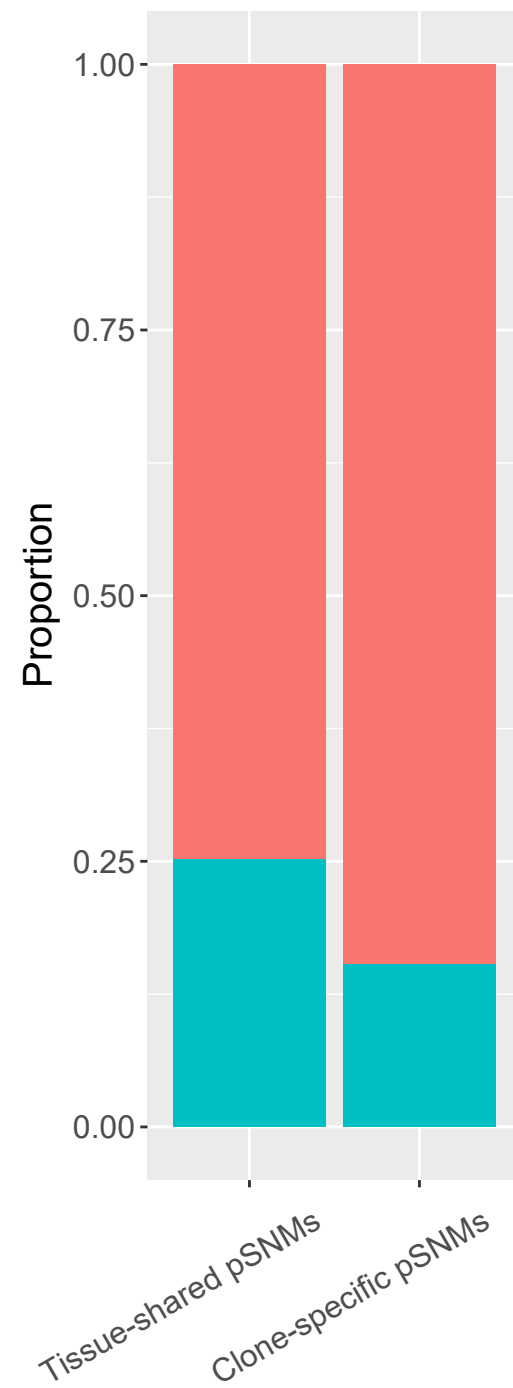

Repressed chromatin

Transcribed chromatin

Supplement: S13 Fig — The pSNMs shared across multiple brain regions or non-brain tissues showed significantly higher proportion of transcribed chromatin status than those specifically present in the clone of neuronal progenitor cells. (PDF) [file pgen.1007395.s013.pdf]

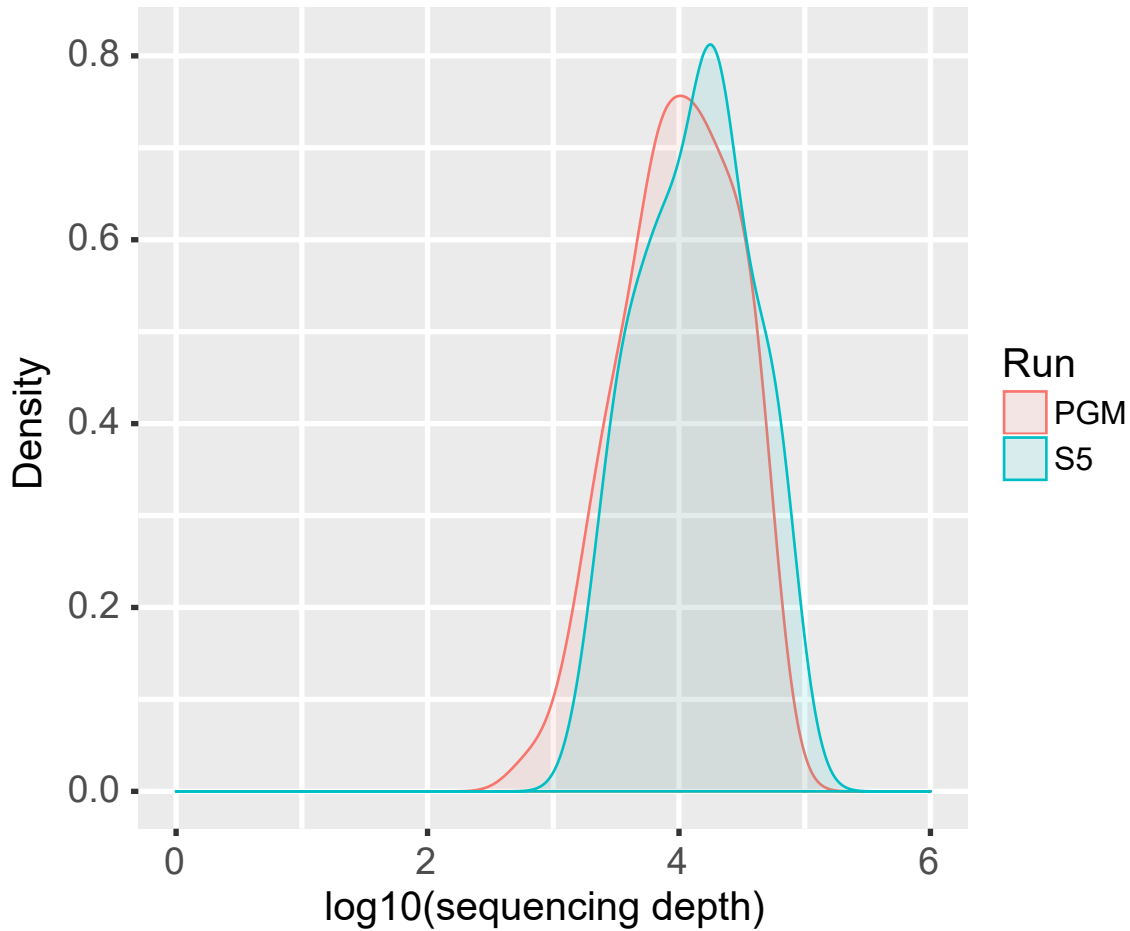

Supplement: S16 Fig — The red and blue curves denote sequencing runs of the same library performed using an Ion Torrent PGM and an Ion S5 XL, respectively. (PDF) [file pgen.1007395.s016.pdf]
