# Supplementary material for: Distinctive types of postzygotic single-nucleotide mosaicisms in healthy individuals revealed by genome-wide profiling of multiple organs
Source: PLoS Genet. 2018 May 15;14(5):e1007395. doi: 10.1371/journal.pgen.1007395 (PMC5969758; doi:10.1371/journal.pgen.1007395)
Supplement: S3 Fig — ACC1 and ACC4 are two unrelated individuals that served as negative controls. The mutant allele fractions were assessed by PGM Amplicon Sequencing of Mosaicism (PASM). Red, green, and blue colors denote heterozygous, mosaic, and reference-homozygous genotypes, respectively. (PDF) [file pgen.1007395.s003.pdf]

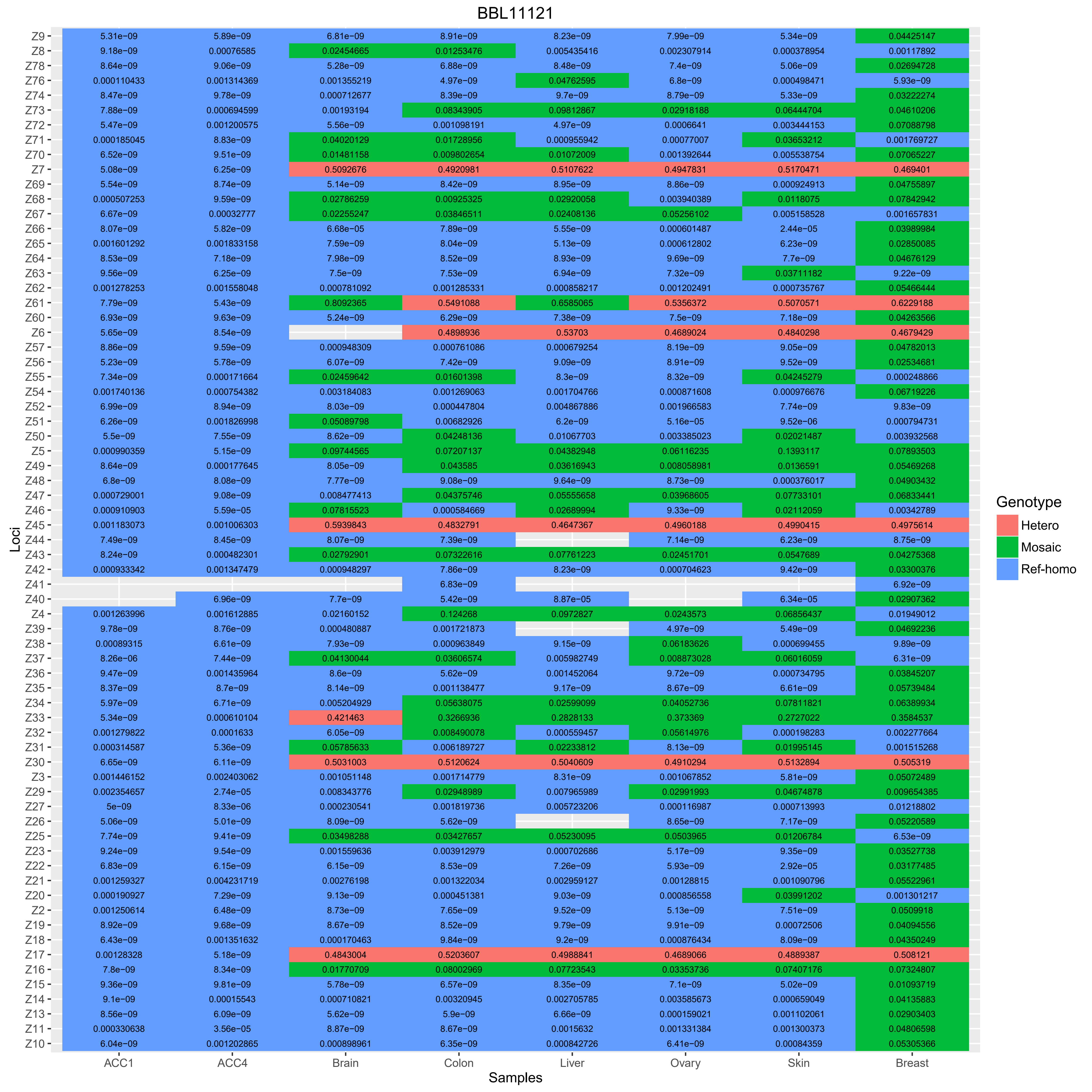
BBL11121
Loci
Samples
ACC1
ACC4
Brain
Colon
Liver
Ovary
Skin
Breast
Genotype
Hetero
Mosaic
Ref-homo
Z9 5.31e-09 5.89e-09 6.81e-09 8.91e-09 8.23e-09 7.99e-09 5.34e-09 0.04425147
Z8 9.18e-09 0.00076585 0.02454665 0.01253476 0.005435416 0.002307914 0.000378954 0.00117892
Z78 8.64e-09 9.06e-09 5.28e-09 6.88e-09 8.48e-09 7.4e-09 5.06e-09 0.02694728
Z76 0.000110433 0.001314369 0.001355219 4.97e-09 0.04762595 6.8e-09 0.000498471 5.93e-09
Z74 8.47e-09 9.78e-09 0.000712677 8.39e-09 9.7e-09 8.79e-09 5.33e-09 0.03222274
Z73 7.88e-09 0.000694599 0.00193194 0.08343905 0.09812867 0.02918188 0.06444704 0.04610206
Z72 5.47e-09 0.001200575 5.56e-09 0.001098191 4.97e-09 0.0006641 0.003444153 0.07088798
Z71 0.000185045 8.83e-09 0.04020129 0.01728956 0.000955942 0.00077007 0.03653212 0.001769727
Z70 6.52e-09 9.51e-09 0.01481158 0.009802654 0.01072009 0.001392644 0.005538754 0.07065227
Z7 5.08e-09 6.25e-09 0.5092676 0.4920981 0.5107622 0.4947831 0.5170471 0.469401
Z69 5.54e-09 8.74e-09 5.14e-09 8.42e-09 8.95e-09 8.86e-09 0.000924913 0.04755897
Z68 0.000507253 9.59e-09 0.02786259 0.00925325 0.02920058 0.003940389 0.0118075 0.07842942
Z67 6.67e-09 0.00032777 0.02255247 0.03846511 0.02408136 0.05256102 0.005158528 0.001657831
Z66 8.07e-09 5.82e-09 6.68e-05 7.89e-09 5.55e-09 0.000601487 2.44e-05 0.03989984
Z65 0.001601292 0.001833158 7.59e-09 8.04e-09 5.13e-09 0.000612802 6.23e-09 0.02850085
Z64 8.53e-09 7.18e-09 7.98e-09 8.52e-09 8.93e-09 9.69e-09 7.7e-09 0.04676129
Z63 9.56e-09 6.25e-09 7.5e-09 7.53e-09 6.94e-09 7.32e-09 0.03711182 9.22e-09
Z62 0.001278253 0.001558048 0.000781092 0.001285331 0.000858217 0.001202491 0.000735767 0.05466444
Z61 7.79e-09 5.43e-09 0.8092365 0.5491088 0.6585065 0.5356372 0.5070571 0.6229188
Z60 6.93e-09 9.63e-09 5.24e-09 6.29e-09 7.38e-09 7.5e-09 7.18e-09 0.04263566
Z6 5.65e-09 8.54e-09 0.4898936 0.53703 0.4689024 0.4840298 0.4679429
Z57 8.86e-09 9.59e-09 0.000948309 0.000761086 0.000679254 8.19e-09 9.05e-09 0.04782013
Z56 5.23e-09 5.78e-09 6.07e-09 7.42e-09 9.09e-09 8.91e-09 9.52e-09 0.02534681
Z55 7.34e-09 0.000171664 0.02459642 0.01601398 8.3e-09 8.32e-09 0.04245279 0.000248866
Z54 0.001740136 0.000754382 0.003184083 0.001269063 0.001704766 0.000871608 0.000976676 0.06719226
Z52 6.99e-09 8.94e-09 8.03e-09 0.000447804 0.004867886 0.001966583 7.74e-09 9.83e-09
Z51 6.26e-09 0.001826998 0.05089798 0.00682926 6.2e-09 5.16e-05 9.52e-06 0.000794731
Z50 5.5e-09 7.55e-09 8.62e-09 0.04248136 0.01067703 0.003385023 0.02021487 0.003932568
Z5 0.000990359 5.15e-09 0.09744565 0.07207137 0.04382948 0.06116235 0.1393117 0.07893503
Z49 8.64e-09 0.000177645 8.05e-09 0.043585 0.03616943 0.008058981 0.0136591 0.05469268
Z48 6.8e-09 8.08e-09 7.77e-09 9.08e-09 9.64e-09 8.73e-09 0.000376017 0.04903432
Z47 0.000729001 9.08e-09 0.008477413 0.04375746 0.05555658 0.03968605 0.07733101 0.06833441
Z46 0.000910903 5.59e-05 0.07815523 0.000584669 0.02689994 9.33e-09 0.02112059 0.00342789
Z45 0.001183073 0.001006303 0.5939843 0.4832791 0.4647367 0.4960188 0.4990415 0.4975614
Z44 7.49e-09 8.45e-09 8.07e-09 7.39e-09 7.14e-09 6.23e-09 8.75e-09
Z43 8.24e-09 0.000482301 0.02792901 0.07322616 0.07761223 0.02451701 0.0547689 0.04275368
Z42 0.000933342 0.001347479 0.000948297 7.86e-09 8.23e-09 0.000704623 9.42e-09 0.03300376
Z41 6.83e-09 6.92e-09
Z40 6.96e-09 7.7e-09 5.42e-09 8.87e-05 6.34e-05 0.02907362
Z4 0.001263996 0.001612885 0.02160152 0.124268 0.0972827 0.0243573 0.06856437 0.01949012
Z39 9.78e-09 8.76e-09 0.000480887 0.001721873 4.97e-09 5.49e-09 0.04692236
Z38 0.00089315 6.61e-09 7.93e-09 0.000963849 9.15e-09 0.06183626 0.000699455 9.89e-09
Z37 8.26e-06 7.44e-09 0.04130044 0.03606574 0.005982749 0.008873028 0.06016059 6.31e-09
Z36 9.47e-09 0.001435964 8.6e-09 5.62e-09 0.001452064 9.72e-09 0.000734795 0.03845207
Z35 8.37e-09 8.7e-09 8.14e-09 0.001138477 9.17e-09 8.67e-09 6.61e-09 0.05739484
Z34 5.97e-09 6.71e-09 0.005204929 0.05638075 0.02599099 0.04052736 0.07811821 0.06389934
Z33 5.34e-09 0.000610104 0.421463 0.3266936 0.2828133 0.373369 0.2727022 0.3584537
Z32 0.001279822 0.0001633 6.05e-09 0.008490078 0.000559457 0.05614976 0.000198283 0.002277664
Z31 0.000314587 5.36e-09 0.05785633 0.006189727 0.02233812 8.13e-09 0.01995145 0.001515268
Z30 6.65e-09 6.11e-09 0.5031003 0.5120624 0.5040609 0.4910294 0.5132894 0.505319
Z3 0.001446152 0.002403062 0.001051148 0.001714779 8.31e-09 0.001067852 5.81e-09 0.05072489
Z29 0.002354657 2.74e-05 0.008343776 0.02948989 0.007965989 0.02991993 0.04674878 0.009654385
Z27 5e-09 8.33e-06 0.000230541 0.001819736 0.005723206 0.000116987 0.000713993 0.01218802
Z26 5.06e-09 5.01e-09 8.09e-09 5.62e-09 8.65e-09 7.17e-09 0.05220589
Z25 7.74e-09 9.41e-09 0.03498288 0.03427657 0.05230095 0.0503965 0.01206784 6.53e-09
Z23 9.24e-09 9.54e-09 0.001559636 0.003912979 0.000702686 5.17e-09 9.35e-09 0.03527738
Z22 6.83e-09 6.15e-09 6.15e-09 8.53e-09 7.26e-09 5.93e-09 2.92e-05 0.03177485
Z21 0.001259327 0.004231719 0.00276198 0.001322034 0.002959127 0.00128815 0.001090796 0.05522961
Z20 0.000190927 7.29e-09 9.13e-09 0.000451381 9.03e-09 0.000856558 0.03991202 0.001301217
Z2 0.001250614 6.48e-09 8.73e-09 7.65e-09 9.52e-09 5.13e-09 7.51e-09 0.0509918
Z19 8.92e-09 9.68e-09 8.67e-09 8.52e-09 9.79e-09 9.91e-09 0.00072506 0.04094556
Z18 6.43e-09 0.001351632 0.000170463 9.84e-09 9.2e-09 0.000876434 8.09e-09 0.04350249
Z17 0.00128328 5.18e-09 0.4843004 0.5203607 0.4988841 0.4689066 0.4889387 0.508121
Z16 7.8e-09 8.34e-09 0.01770709 0.08002969 0.07723543 0.03353736 0.07407176 0.07324807
Z15 9.36e-09 9.81e-09 5.78e-09 6.57e-09 8.35e-09 7.1e-09 5.02e-09 0.01093719
Z14 9.1e-09 0.00015543 0.000710821 0.00320945 0.002705785 0.003585673 0.000659049 0.04135883
Z13 8.56e-09 6.09e-09 5.62e-09 5.9e-09 6.66e-09 0.000159021 0.001102061 0.02903403
Z11 0.000330638 3.56e-05 8.87e-09 8.67e-09 0.0015632 0.001331384 0.001300373 0.04806598
Z10 6.04e-09 0.001202865 0.000898961 6.35e-09 0.000842726 6.41e-09 0.00084359 0.05305366
