# Supplementary material for: Distinctive types of postzygotic single-nucleotide mosaicisms in healthy individuals revealed by genome-wide profiling of multiple organs
Source: PLoS Genet. 2018 May 15;14(5):e1007395. doi: 10.1371/journal.pgen.1007395 (PMC5969758; doi:10.1371/journal.pgen.1007395)
Supplement: S14 Fig — Both types of embryonic pSNMs showed predominant C>T mutations and elevated mutation rate in CpG sites. (PDF) [file pgen.1007395.s014.pdf]

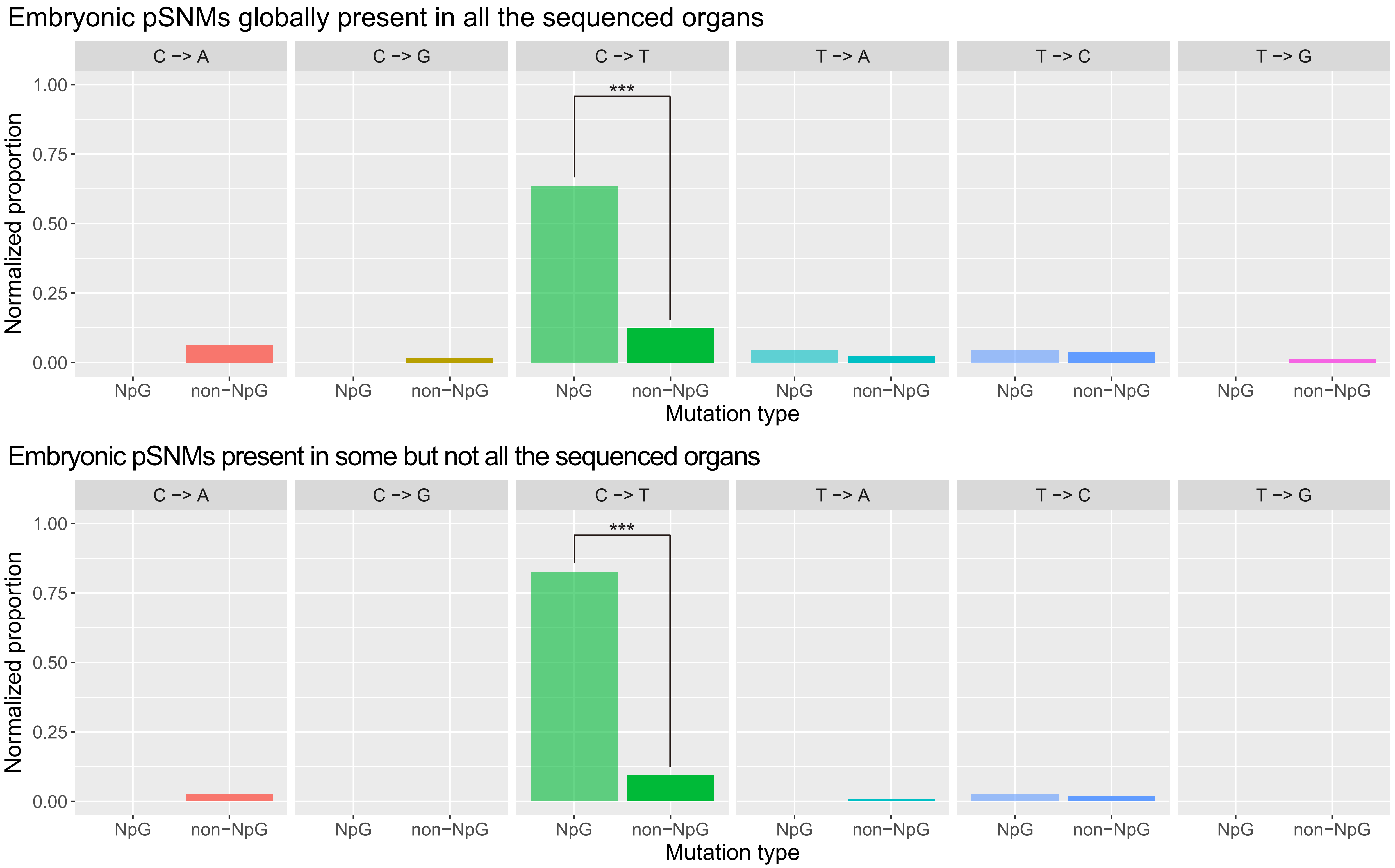

Embryonic pSNMs globally present in all the sequenced organs
C -> A
C -> G
C -> T
T -> A
T -> C
T -> G
***
Normalized proportion
1.00
0.75
0.50
0.25
0.00
NpG
non−NpG
Mutation type
Embryonic pSNMs present in some but not all the sequenced organs
C -> A
C -> G
C -> T
T -> A
T -> C
T -> G
***
Normalized proportion
1.00
0.75
0.50
0.25
0.00
NpG
non−NpG
Mutation type
