# Supplementary material for: Distinctive types of postzygotic single-nucleotide mosaicisms in healthy individuals revealed by genome-wide profiling of multiple organs
Source: PLoS Genet. 2018 May 15;14(5):e1007395. doi: 10.1371/journal.pgen.1007395 (PMC5969758; doi:10.1371/journal.pgen.1007395)
Supplement: S15 Fig — Both types of embryonic pSNMs demonstrated the similar enrichment for early-replicating regions. (PDF) [file pgen.1007395.s015.pdf]

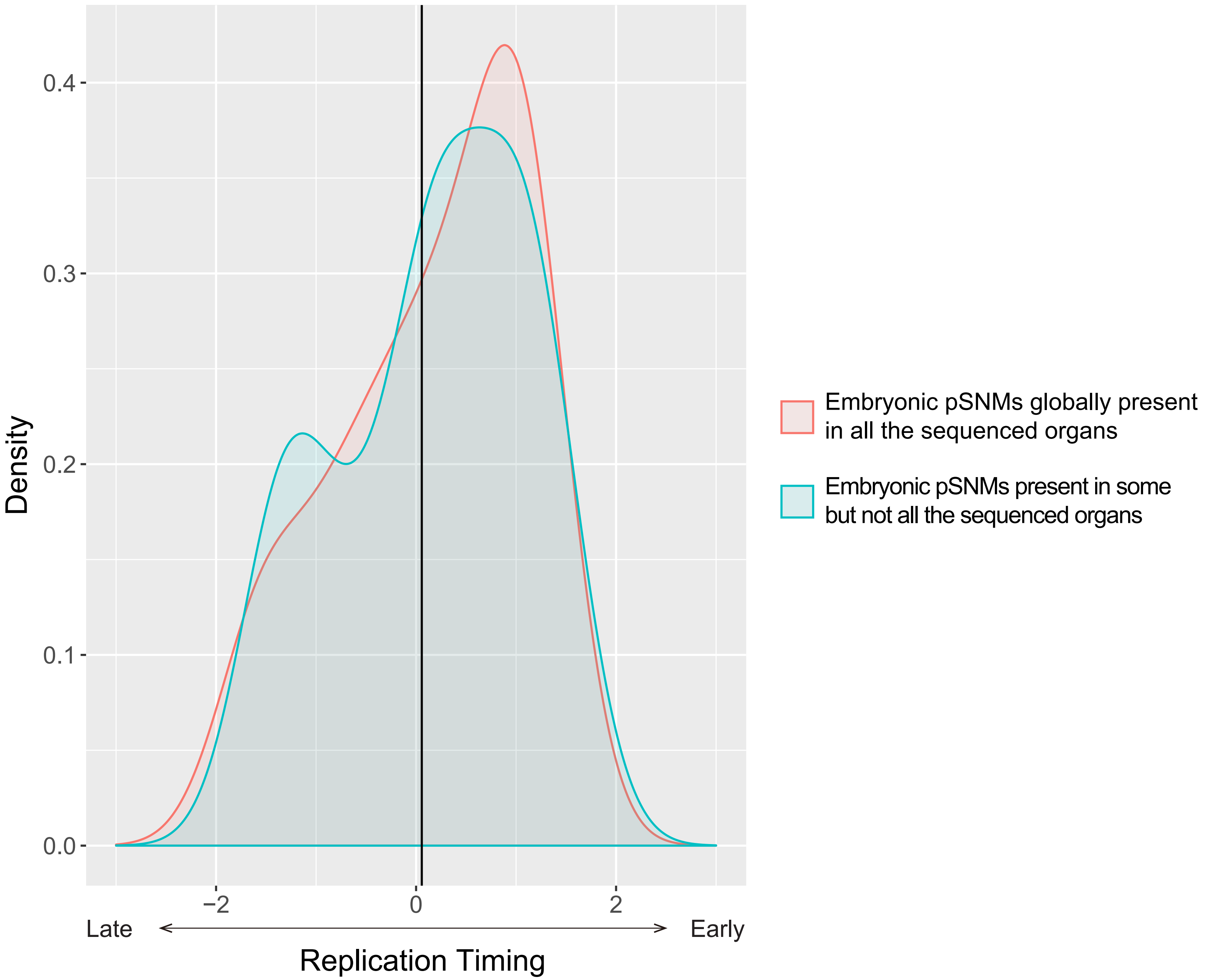

Density
0.4
0.3
0.2
0.1
0.0
−2
0
2
Late
Early
Replication Timing
Embryonic pSNMs globally present in all the sequenced organs
Embryonic pSNMs present in some but not all the sequenced organs
