# Supplementary material for: Distinctive types of postzygotic single-nucleotide mosaicisms in healthy individuals revealed by genome-wide profiling of multiple organs
Source: PLoS Genet. 2018 May 15;14(5):e1007395. doi: 10.1371/journal.pgen.1007395 (PMC5969758; doi:10.1371/journal.pgen.1007395)
Supplement: S17 Fig — Sample IDs starting with “b”, “c”, “l”, “p”, and “s” denote multiple samples obtained from brain, colon, liver, prostate, and skin of BBL1100C, respectively. Triple technical replicates of PGM Amplicon Sequencing of Mosaicism (PASM) were performed for the V18 pSNM of each sample. The inter-organ and intra-organ variations are dramatically larger than the variation observed between technical replicates. (PDF) [file pgen.1007395.s017.pdf]

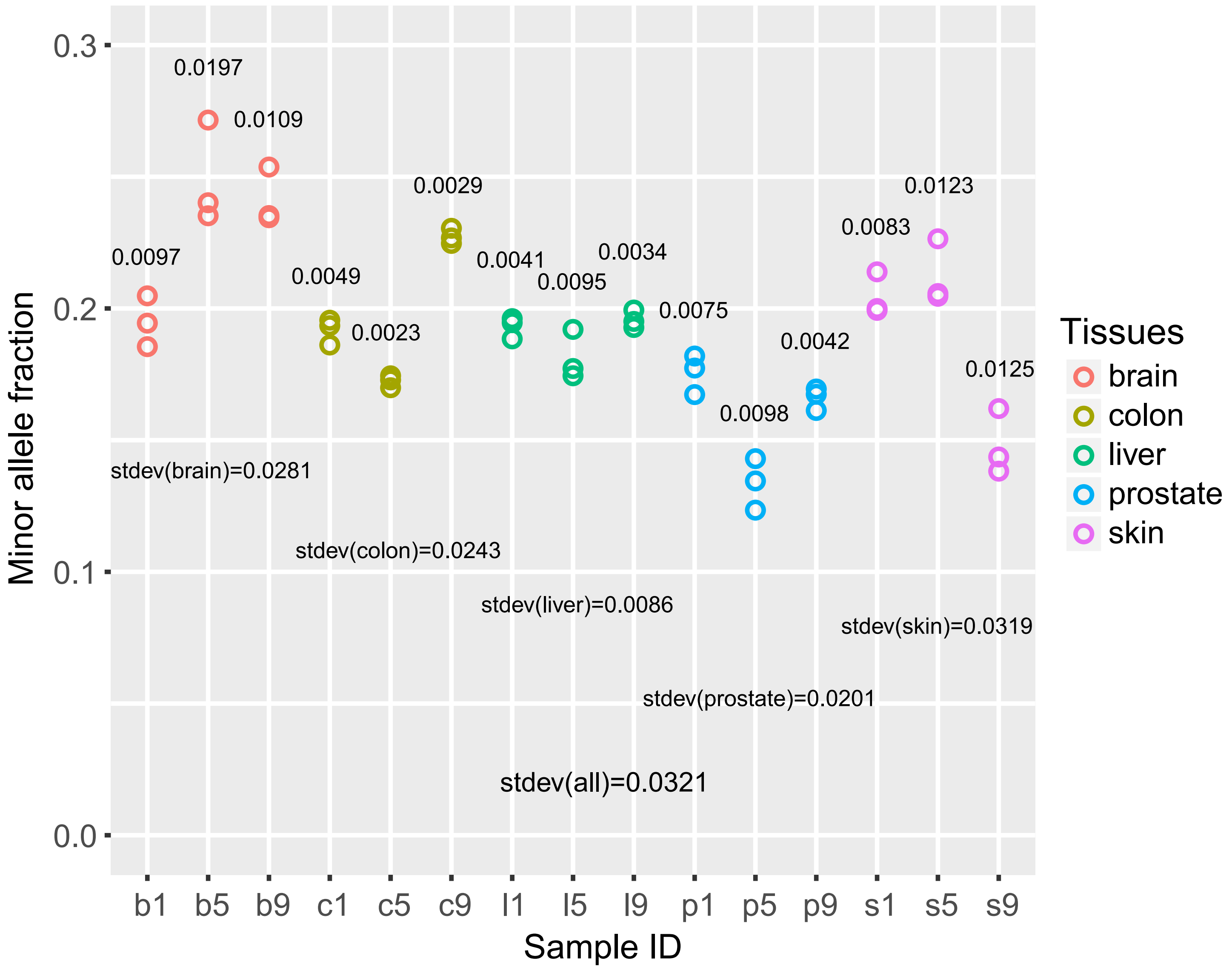

0.3
0.2
0.1
0.0
Minor allele fraction
0.0197
0.0109
0.0097
0.0049
0.0023
0.0029
0.0041
0.0095
0.0034
0.0075
0.0098
0.0042
0.0083
0.0123
0.0125
stdev(brain)=0.0281
stdev(colon)=0.0243
stdev(liver)=0.0086
stdev(skin)=0.0319
stdev(prostate)=0.0201
stdev(all)=0.0321
b1 b5 b9 c1 c5 c9 l1 l5 l9 p1 p5 p9 s1 s5 s9
Sample ID
Tissues
brain
colon
liver
prostate
skin
