# Supplementary material for: Distinctive types of postzygotic single-nucleotide mosaicisms in healthy individuals revealed by genome-wide profiling of multiple organs
Source: PLoS Genet. 2018 May 15;14(5):e1007395. doi: 10.1371/journal.pgen.1007395 (PMC5969758; doi:10.1371/journal.pgen.1007395)
Supplement: S1 Table — (DOC) [file pgen.1007395.s018.doc]

**S1 Table. Copy number estimation of candidate mosaic sites.**

| **ID** | **Pos** | **Individual** | **Sample with mosaic genotype** | **Allele fraction** | **Copy number** |
| --- | --- | --- | --- | --- | --- |
| **L2** | 1:26769815 | BBL1100C | Colon,Prostate | 0.055 | 2.139 |
| **V25** | 1:114973790 | BBL1100C | Brain,Colon,Liver,Prostate,Skin | 0.020 | 2.102 |
| **V2** | 1:224876814 | BBL1100C | Brain,Colon,Liver,Prostate,Skin | 0.170 | 1.835 |
| **V5** | 2:135722990 | BBL1100C | Brain,Liver,Prostate | 0.024 | 2.149 |
| **V30** | 2:228568980 | BBL1100C | Brain,Colon,Liver,Prostate,Skin | 0.057 | 1.940 |
| **L19** | 3:12394900 | BBL1100C | Prostate | 0.085 | 2.168 |
| **V9** | 4:41634722 | BBL1100C | Brain,Colon,Liver,Prostate,Skin | 0.297 | 2.031 |
| **L27** | 4:154773423 | BBL1100C | Skin | 0.028 | 1.987 |
| **V10** | 4:181048637 | BBL1100C | Brain,Colon,Liver,Prostate,Skin | 0.142 | 2.041 |
| **V31** | 5:64603697 | BBL1100C | Brain,Colon,Liver,Skin | 0.036 | 2.043 |
| **V33** | 5:125465949 | BBL1100C | Liver,Skin | 0.013 | 1.847 |
| **V35** | 6:164101476 | BBL1100C | Brain,Colon,Prostate,Skin | 0.023 | 2.259 |
| **V18** | 11:134115864 | BBL1100C | Brain,Colon,Liver,Prostate,Skin | 0.216 | 2.075 |
| **V19** | 14:37786847 | BBL1100C | Brain,Prostate,Skin | 0.033 | 1.961 |
| **L71** | 20:22982519 | BBL1100C | Skin | 0.044 | 1.990 |
| **V23** | 20:40919410 | BBL1100C | Brain,Colon,Liver,Prostate,Skin | 0.082 | 1.966 |
| **Z2** | 1:50640673 | BBL11121 | Breast | 0.051 | 1.961 |
| **Z3** | 1:99953413 | BBL11121 | Breast | 0.051 | 1.960 |
| **Z4** | 1:234747291 | BBL11121 | Liver,Colon,Ovary,Skin,Breast | 0.082 | 2.053 |
| **Z5** | 1:241471556 | BBL11121 | Brain,Liver,Colon,Ovary,Skin,Breast | 0.089 | 2.084 |
| **Z31** | 2:3260480 | BBL11121 | Brain,Liver,Skin,Breast | 0.030 | 2.064 |
| **Z32** | 2:3913156 | BBL11121 | Colon,Ovary | 0.032 | 1.999 |
| **Z34** | 2:47722338 | BBL11121 | Liver,Colon,Ovary,Skin,Breast | 0.061 | 1.977 |
| **Z35** | 2:126487527 | BBL11121 | Breast | 0.057 | 2.074 |
| **Z36** | 2:226710617 | BBL11121 | Breast | 0.038 | 2.041 |
| **Z37** | 2:227430472 | BBL11121 | Brain,Colon,Ovary,Skin,Breast | 0.034 | 1.991 |
| **Z38** | 2:236800392 | BBL11121 | Ovary | 0.062 | 1.790 |
| **Z46** | 3:28336801 | BBL11121 | Brain,Liver,Skin,Breast | 0.035 | 2.024 |
| **Z47** | 3:31252080 | BBL11121 | Liver,Colon,Ovary,Skin,Breast | 0.066 | 1.991 |
| **Z48** | 3:36701049 | BBL11121 | Breast | 0.049 | 1.855 |
| **Z49** | 3:115320238 | BBL11121 | Liver,Colon,Ovary,Skin,Breast | 0.049 | 2.044 |
| **Z50** | 3:165204247 | BBL11121 | Colon,Skin,Breast | 0.022 | 1.958 |
| **Z51** | 3:175619792 | BBL11121 | Brain,Breast | 0.037 | 2.080 |
| **Z54** | 4:180446403 | BBL11121 | Breast | 0.067 | 1.993 |
| **Z55** | 5:88341379 | BBL11121 | Brain,Colon,Skin | 0.028 | 2.062 |
| **Z56** | 6:73468560 | BBL11121 | Breast | 0.025 | 1.991 |
| **Z57** | 6:80434788 | BBL11121 | Breast | 0.048 | 1.901 |
| **Z60** | 6:143020735 | BBL11121 | Breast | 0.043 | 1.972 |
| **Z62** | 6:170547487 | BBL11121 | Breast | 0.055 | 1.913 |
| **Z63** | 7:1425148 | BBL11121 | Skin | 0.037 | 2.089 |
| **Z64** | 7:68358382 | BBL11121 | Breast | 0.047 | 1.950 |
| **Z65** | 7:79880023 | BBL11121 | Breast | 0.029 | 2.088 |
| **Z66** | 7:103982586 | BBL11121 | Breast | 0.040 | 2.082 |
| **Z67** | 8:5881031 | BBL11121 | Brain,Liver,Colon,Ovary,Breast | 0.029 | 1.939 |
| **Z68** | 8:61243836 | BBL11121 | Brain,Liver,Colon,Skin,Breast | 0.036 | 2.259 |
| **Z69** | 8:100642472 | BBL11121 | Breast | 0.047 | 2.005 |
| **Z70** | 8:109523811 | BBL11121 | Brain,Liver,Colon,Breast | 0.033 | 1.941 |
| **Z71** | 8:119263974 | BBL11121 | Brain,Colon,Skin | 0.031 | 1.979 |
| **Z72** | 8:131927641 | BBL11121 | Breast | 0.037 | 2.142 |
| **Z73** | 9:28463213 | BBL11121 | Liver,Colon,Ovary,Skin,Breast | 0.076 | 1.980 |
| **Z74** | 9:28943014 | BBL11121 | Breast | 0.032 | 2.034 |
| **Z8** | 10:68687696 | BBL11121 | Brain,Colon,Breast | 0.025 | 2.094 |
| **Z9** | 10:120489728 | BBL11121 | Breast | 0.044 | 1.881 |
| **Z10** | 11:131717828 | BBL11121 | Breast | 0.053 | 1.999 |
| **Z11** | 11:132113942 | BBL11121 | Breast | 0.048 | 2.028 |
| **Z13** | 12:79409138 | BBL11121 | Breast | 0.029 | 1.917 |
| **Z14** | 12:130128978 | BBL11121 | Breast | 0.035 | 1.875 |
| **Z15** | 13:23780127 | BBL11121 | Breast | 0.011 | 1.951 |
| **Z16** | 13:23910741 | BBL11121 | Brain,Liver,Colon,Ovary,Skin,Breast | 0.073 | 2.149 |
| **Z18** | 13:79395067 | BBL11121 | Breast | 0.044 | 1.967 |
| **Z19** | 13:82873560 | BBL11121 | Breast | 0.041 | 2.160 |
| **Z20** | 13:104700276 | BBL11121 | Skin | 0.040 | 2.106 |
| **Z21** | 14:50549268 | BBL11121 | Breast | 0.055 | 1.986 |
| **Z22** | 14:66089975 | BBL11121 | Breast | 0.032 | 2.038 |
| **Z23** | 14:74665394 | BBL11121 | Breast | 0.022 | 1.934 |
| **Z25** | 17:2084756 | BBL11121 | Brain,Liver,Colon,Ovary,Skin,Breast | 0.032 | 1.979 |
| **Z26** | 17:7077804 | BBL11121 | Breast | 0.052 | 1.913 |
| **Z27** | 17:52228988 | BBL11121 | Breast | 0.047 | 1.935 |
| **Z29** | 18:75697941 | BBL11121 | Colon,Ovary,Skin,Breast | 0.032 | 1.993 |
| **Z39** | 20:6174493 | BBL11121 | Breast | 0.047 | 2.083 |
| **Z40** | 20:15885200 | BBL11121 | Breast | 0.029 | 1.957 |
| **Z42** | 21:24895983 | BBL11121 | Breast | 0.033 | 1.927 |
| **Z43** | 21:26729459 | BBL11121 | Brain,Liver,Colon,Ovary,Skin,Breast | 0.067 | 1.981 |
| **Z76** | X:20373572 | BBL11121 | Liver | 0.048 | 1.889 |
| **Z78** | X:150909213 | BBL11121 | Breast | 0.027 | 2.180 |
| **Q1** | 1:22225123 | BBLC1013 | Artery,Brain,Colon,Liver,Prostate,Skin | 0.048 | 1.980 |
| **Q2** | 1:64465989 | BBLC1013 | Artery,Brain,Colon,Liver,Prostate,Skin | 0.040 | 1.968 |
| **Q4** | 2:875456 | BBLC1013 | Liver | 0.069 | 2.112 |
| **Q5** | 2:38366144 | BBLC1013 | Prostate | 0.039 | 2.090 |
| **Q7** | 3:61685928 | BBLC1013 | Artery,Brain,Colon,Liver,Prostate,Skin | 0.262 | **2.792** |
| **Q9** | 3:168112730 | BBLC1013 | Artery | 0.032 | 1.929 |
| **Q12** | 4:67061519 | BBLC1013 | Artery,Brain,Colon,Liver,Prostate,Skin | 0.187 | 1.982 |
| **Q13** | 4:67129912 | BBLC1013 | Artery,Brain,Colon,Liver,Skin | 0.069 | 2.194 |
| **Q14** | 4:147210434 | BBLC1013 | Prostate | 0.021 | 2.234 |
| **Q17** | 6:69672804 | BBLC1013 | Prostate | 0.042 | 2.101 |
| **Q20** | 7:30959565 | BBLC1013 | Artery,Brain,Skin | 0.014 | 1.930 |
| **Q23** | 8:15332347 | BBLC1013 | Artery,Brain,Liver,Prostate,Skin | 0.167 | 1.956 |
| **Q26** | 12:118603305 | BBLC1013 | Artery,Brain,Liver,Prostate | 0.044 | 2.096 |
| **Q30** | 16:77628019 | BBLC1013 | Artery,Brain,Colon,Liver,Prostate,Skin | 0.076 | 2.020 |
| **Q31** | 17:78776823 | BBLC1013 | Artery,Brain,Colon,Liver,Prostate,Skin | 0.023 | 1.751 |
| **Q32** | 18:74589625 | BBLC1013 | Liver | 0.137 | 2.027 |
| **Q35** | 22:24132092 | BBLC1013 | Artery,Brain,Prostate | 0.027 | 1.970 |
| **P1** | 1:49035302 | BBLD1005 | Liver | 0.020 | 2.055 |
| **P4** | 1:244316714 | BBLD1005 | Artery,Brain,Colon,Liver,Skin | 0.051 | 2.050 |
| **P5** | 2:105741925 | BBLD1005 | Liver | 0.021 | 2.132 |
| **P7** | 2:181549566 | BBLD1005 | Liver | 0.021 | 2.236 |
| **P11** | 4:56210847 | BBLD1005 | Artery,Colon,Liver,Skin | 0.035 | 2.020 |
| **P13** | 4:82184505 | BBLD1005 | Liver | 0.014 | 2.094 |
| **P14** | 4:97078131 | BBLD1005 | Liver | 0.014 | 2.013 |
| **P15** | 4:97707271 | BBLD1005 | Liver | 0.022 | 1.938 |
| **P16** | 5:6810959 | BBLD1005 | Liver | 0.019 | 2.055 |
| **P17** | 5:18840767 | BBLD1005 | Liver | 0.022 | 2.002 |
| **P18** | 5:38097742 | BBLD1005 | Liver | 0.040 | 2.087 |
| **P19** | 5:59002752 | BBLD1005 | Artery,Brain,Colon,Liver,Skin | 0.145 | 1.985 |
| **P20** | 5:108791612 | BBLD1005 | Liver | 0.027 | 1.952 |
| **P21** | 6:9117410 | BBLD1005 | Liver | 0.015 | 1.951 |
| **P22** | 6:113444094 | BBLD1005 | Liver | 0.028 | 2.060 |
| **P24** | 6:139842988 | BBLD1005 | Liver | 0.012 | 1.939 |
| **P25** | 6:143426551 | BBLD1005 | Brain,Liver,Skin | 0.016 | 1.992 |
| **P26** | 7:101860696 | BBLD1005 | Liver | 0.023 | 1.962 |
| **P27** | 7:143002185 | BBLD1005 | Brain | 0.024 | 1.949 |
| **P28** | 8:13105971 | BBLD1005 | Liver | 0.027 | 2.185 |
| **P29** | 8:62581864 | BBLD1005 | Liver | 0.020 | 1.993 |
| **P30** | 8:99610896 | BBLD1005 | Liver | 0.036 | 2.146 |
| **P33** | 10:13562293 | BBLD1005 | Colon | 0.015 | 2.033 |
| **P34** | 10:36073362 | BBLD1005 | Liver | 0.015 | 2.010 |
| **P35** | 10:80628845 | BBLD1005 | Artery,Brain,Colon,Liver,Skin | 0.309 | **2.803** |
| **P36** | 10:85183194 | BBLD1005 | Artery,Brain,Colon,Liver,Skin | 0.160 | 2.166 |
| **P37** | 11:1391515 | BBLD1005 | Liver | 0.037 | 1.834 |
| **P38** | 11:76807693 | BBLD1005 | Artery,Brain,Colon,Liver,Skin | 0.031 | 2.041 |
| **P39** | 11:106871001 | BBLD1005 | Liver | 0.024 | 2.079 |
| **P40** | 12:2324301 | BBLD1005 | Liver | 0.010 | 1.986 |
| **P42** | 12:26901510 | BBLD1005 | Liver | 0.065 | 2.036 |
| **P43** | 12:74691796 | BBLD1005 | Liver | 0.024 | 2.221 |
| **P44** | 12:87993265 | BBLD1005 | Liver | 0.025 | 2.065 |
| **P45** | 13:55948090 | BBLD1005 | Liver | 0.032 | 2.040 |
| **P48** | 14:21736571 | BBLD1005 | Liver | 0.021 | 1.780 |
| **P49** | 14:82146609 | BBLD1005 | Liver | 0.017 | 2.022 |
| **P50** | 14:96305274 | BBLD1005 | Artery,Brain,Colon,Liver,Skin | 0.152 | 1.968 |
| **P51** | 15:25350138 | BBLD1005 | Liver | 0.042 | 1.977 |
| **P52** | 15:94590035 | BBLD1005 | Liver | 0.026 | 2.029 |
| **P53** | 16:3639261 | BBLD1005 | Brain,Colon,Liver | 0.032 | 2.149 |
| **P54** | 16:18814565 | BBLD1005 | Liver | 0.021 | 1.842 |
| **P56** | 18:12104914 | BBLD1005 | Liver | 0.023 | 1.974 |
| **P58** | 18:38513947 | BBLD1005 | Liver | 0.022 | 2.026 |
| **P59** | 18:53608260 | BBLD1005 | Liver | 0.021 | 2.145 |
| **P62** | 20:9305925 | BBLD1005 | Liver | 0.038 | 2.083 |
| **P63** | 20:19541238 | BBLD1005 | Liver | 0.014 | 2.051 |
| **P64** | 21:46529741 | BBLD1005 | Liver | 0.028 | 1.856 |
| **P65** | X:7442005 | BBLD1005 | Artery,Colon,Liver,Skin | 0.152 | 0.954 |
| **P66** | X:22516173 | BBLD1005 | Liver | 0.012 | 1.004 |
| **P67** | X:25272450 | BBLD1005 | Liver | 0.047 | 1.031 |
| **P69** | X:117758307 | BBLD1005 | Liver | 0.011 | 0.970 |
| **P70** | X:125573982 | BBLD1005 | Liver | 0.032 | 0.979 |
| **P71** | X:127329197 | BBLD1005 | Liver | 0.069 | 1.035 |
| **P72** | X:132692389 | BBLD1005 | Liver | 0.020 | 0.987 |
| **P73** | X:142880750 | BBLD1005 | Liver | 0.056 | 1.027 |
| **N35** | 1:5144704 | BBLD1010 | Colon,Prostate | 0.097 | 1.975 |
| **N36** | 3:74385683 | BBLD1010 | Prostate | 0.076 | 1.995 |
| **N3** | 3:126380038 | BBLD1010 | Brain,Liver | 0.015 | 1.838 |
| **N5** | 4:22712686 | BBLD1010 | Prostate | 0.087 | 1.948 |
| **N7** | 6:167609855 | BBLD1010 | Brain,Colon,Liver,Prostate,Skin | 0.246 | 2.016 |
| **N8** | 7:49883392 | BBLD1010 | Skin | 0.017 | 1.976 |
| **N10** | 8:50367950 | BBLD1010 | Prostate | 0.098 | 2.061 |
| **N11** | 8:92360228 | BBLD1010 | Prostate | 0.090 | 1.975 |
| **N13** | 9:33270489 | BBLD1010 | Brain,Colon,Liver,Skin | 0.085 | 2.094 |
| **N14** | 9:93652243 | BBLD1010 | Skin | 0.049 | 1.929 |
| **N15** | 9:117788988 | BBLD1010 | Brain,Colon,Liver,Prostate,Skin | 0.206 | 1.883 |
| **N19** | 12:2027542 | BBLD1010 | Prostate | 0.196 | 1.985 |
| **N20** | 13:96860919 | BBLD1010 | Skin | 0.042 | 2.107 |
| **N21** | 15:89676236 | BBLD1010 | Brain,Colon,Liver,Prostate,Skin | 0.214 | 1.966 |
| **N22** | 15:94441079 | BBLD1010 | Prostate | 0.064 | 2.096 |
| **N23** | 15:100647704 | BBLD1010 | Liver | 0.024 | 1.896 |
| **N25** | 16:66671706 | BBLD1010 | Prostate | 0.127 | 2.079 |
| **N26** | 17:41441765 | BBLD1010 | Brain,Colon,Liver,Prostate,Skin | 0.288 | **2.587** |
| **N27** | 17:62324984 | BBLD1010 | Colon | 0.102 | 1.981 |
| **N30** | 21:17967785 | BBLD1010 | Prostate | 0.117 | 2.190 |
