# Supplementary material for: Distinctive types of postzygotic single-nucleotide mosaicisms in healthy individuals revealed by genome-wide profiling of multiple organs
Source: PLoS Genet. 2018 May 15;14(5):e1007395. doi: 10.1371/journal.pgen.1007395 (PMC5969758; doi:10.1371/journal.pgen.1007395)
Supplement: S2 Table — (DOC) [file pgen.1007395.s019.doc]

**S2 Table. List of the 164 validated mosaic sites identified from five individuals.**

| **Position** | **Ref** | **Alt** | **Individual** | **Tissue types for mosaic genotype** | **Type** | **Allele fraction** | |
| --- | --- | --- | --- | --- | --- | --- | --- |
|  |  |  |  |  |  | **Mean** | **SD** |
| **1:26769815** | G | A | BBL1100C | Colon,Prostate | Shared | 0.055 | 0.001 |
| **1:114973790** | T | C | BBL1100C | Brain,Colon,Liver,Prostate,Skin | Shared | 0.020 | 0.005 |
| **1:224876814** | G | A | BBL1100C | Brain,Colon,Liver,Prostate,Skin | Shared | 0.170 | 0.053 |
| **2:135722990** | G | A | BBL1100C | Brain,Liver,Prostate | Shared | 0.024 | 0.020 |
| **2:228568980** | T | G | BBL1100C | Brain,Colon,Liver,Prostate,Skin | Shared | 0.057 | 0.045 |
| **3:12394900** | A | G | BBL1100C | Prostate | Unique | 0.085 | NA |
| **4:41634722** | G | A | BBL1100C | Brain,Colon,Liver,Prostate,Skin | Shared | 0.297 | 0.040 |
| **4:154773423** | A | T | BBL1100C | Skin | Unique | 0.028 | NA |
| **4:181048637** | T | C | BBL1100C | Brain,Colon,Liver,Prostate,Skin | Shared | 0.142 | 0.032 |
| **5:64603697** | C | T | BBL1100C | Brain,Colon,Liver,Skin | Shared | 0.036 | 0.022 |
| **5:125465949** | T | C | BBL1100C | Liver,Skin | Shared | 0.013 | 0.006 |
| **6:164101476** | T | C | BBL1100C | Brain,Colon,Prostate,Skin | Shared | 0.023 | 0.008 |
| **11:134115864** | C | A | BBL1100C | Brain,Colon,Liver,Prostate,Skin | Shared | 0.216 | 0.033 |
| **14:37786847** | G | A | BBL1100C | Brain,Prostate,Skin | Shared | 0.033 | 0.022 |
| **20:22982519** | G | A | BBL1100C | Skin | Unique | 0.044 | NA |
| **20:40919410** | T | A | BBL1100C | Brain,Colon,Liver,Prostate,Skin | Shared | 0.082 | 0.027 |
| **1:50640673** | T | C | BBL11121 | Breast | Unique | 0.051 | NA |
| **1:99953413** | T | C | BBL11121 | Breast | Unique | 0.051 | NA |
| **1:234747291** | A | G | BBL11121 | Liver,Colon,Ovary,Skin,Breast | Shared | 0.082 | 0.044 |
| **1:241471556** | A | G | BBL11121 | Brain,Liver,Colon,Ovary,Skin,Breast | Shared | 0.089 | 0.031 |
| **2:3260480** | C | T | BBL11121 | Brain,Liver,Skin,Breast | Shared | 0.030 | 0.019 |
| **2:3913156** | C | T | BBL11121 | Colon,Ovary | Shared | 0.032 | 0.034 |
| **2:47722338** | C | A | BBL11121 | Liver,Colon,Ovary,Skin,Breast | Shared | 0.061 | 0.021 |
| **2:126487527** | G | A | BBL11121 | Breast | Unique | 0.057 | NA |
| **2:226710617** | C | T | BBL11121 | Breast | Unique | 0.038 | NA |
| **2:227430472** | C | T | BBL11121 | Brain,Colon,Ovary,Skin,Breast | Shared | 0.034 | 0.019 |
| **2:236800392** | G | A | BBL11121 | Ovary | Unique | 0.062 | NA |
| **3:28336801** | C | T | BBL11121 | Brain,Liver,Skin,Breast | Shared | 0.035 | 0.030 |
| **3:31252080** | C | A | BBL11121 | Liver,Colon,Ovary,Skin,Breast | Shared | 0.066 | 0.021 |
| **3:36701049** | G | T | BBL11121 | Breast | Unique | 0.049 | NA |
| **3:115320238** | C | T | BBL11121 | Liver,Colon,Ovary,Skin,Breast | Shared | 0.049 | 0.031 |
| **3:165204247** | C | A | BBL11121 | Colon,Skin,Breast | Shared | 0.022 | 0.014 |
| **3:175619792** | C | T | BBL11121 | Brain,Breast | Shared | 0.037 | 0.020 |
| **4:180446403** | A | G | BBL11121 | Breast | Unique | 0.067 | NA |
| **5:88341379** | G | A | BBL11121 | Brain,Colon,Skin | Shared | 0.028 | 0.013 |
| **6:73468560** | G | A | BBL11121 | Breast | Unique | 0.025 | NA |
| **6:80434788** | C | T | BBL11121 | Breast | Unique | 0.048 | NA |
| **6:143020735** | C | T | BBL11121 | Breast | Unique | 0.043 | NA |
| **6:170547487** | T | C | BBL11121 | Breast | Unique | 0.055 | NA |
| **7:1425148** | G | A | BBL11121 | Skin | Unique | 0.037 | NA |
| **7:68358382** | G | C | BBL11121 | Breast | Unique | 0.047 | NA |
| **7:79880023** | A | G | BBL11121 | Breast | Unique | 0.029 | NA |
| **7:103982586** | T | C | BBL11121 | Breast | Unique | 0.040 | NA |
| **8:5881031** | G | A | BBL11121 | Brain,Liver,Colon,Ovary,Breast | Shared | 0.029 | 0.017 |
| **8:61243836** | G | A | BBL11121 | Brain,Liver,Colon,Skin,Breast | Shared | 0.036 | 0.026 |
| **8:100642472** | C | G | BBL11121 | Breast | Unique | 0.047 | NA |
| **8:109523811** | C | T | BBL11121 | Brain,Liver,Colon,Breast | Shared | 0.033 | 0.026 |
| **8:119263974** | G | A | BBL11121 | Brain,Colon,Skin | Shared | 0.031 | 0.012 |
| **8:131927641** | A | G | BBL11121 | Breast | Unique | 0.037 | NA |
| **9:28463213** | G | A | BBL11121 | Liver,Colon,Ovary,Skin,Breast | Shared | 0.076 | 0.031 |
| **9:28943014** | C | G | BBL11121 | Breast | Unique | 0.032 | NA |
| **10:68687696** | C | T | BBL11121 | Brain,Colon,Breast | Shared | 0.025 | 0.013 |
| **10:120489728** | G | C | BBL11121 | Breast | Unique | 0.044 | NA |
| **11:131717828** | A | G | BBL11121 | Breast | Unique | 0.053 | NA |
| **11:132113942** | A | T | BBL11121 | Breast | Unique | 0.048 | NA |
| **12:79409138** | G | T | BBL11121 | Breast | Unique | 0.029 | NA |
| **12:130128978** | G | A | BBL11121 | Breast | Unique | 0.035 | NA |
| **13:23780127** | C | T | BBL11121 | Breast | Unique | 0.011 | NA |
| **13:23910741** | C | T | BBL11121 | Brain,Liver,Colon,Ovary,Skin,Breast | Shared | 0.073 | 0.033 |
| **13:79395067** | A | G | BBL11121 | Breast | Unique | 0.044 | NA |
| **13:82873560** | T | A | BBL11121 | Breast | Unique | 0.041 | NA |
| **13:104700276** | A | G | BBL11121 | Skin | Unique | 0.040 | NA |
| **14:50549268** | T | C | BBL11121 | Breast | Unique | 0.055 | NA |
| **14:66089975** | G | A | BBL11121 | Breast | Unique | 0.032 | NA |
| **14:74665394** | T | C | BBL11121 | Breast | Unique | 0.022 | NA |
| **17:2084756** | G | A | BBL11121 | Brain,Liver,Colon,Ovary,Skin,Breast | Shared | 0.032 | 0.018 |
| **17:7077804** | C | T | BBL11121 | Breast | Unique | 0.052 | NA |
| **17:52228988** | T | C | BBL11121 | Breast | Unique | 0.047 | NA |
| **18:75697941** | A | G | BBL11121 | Colon,Ovary,Skin,Breast | Shared | 0.032 | 0.021 |
| **20:6174493** | A | G | BBL11121 | Breast | Unique | 0.047 | NA |
| **20:15885200** | A | G | BBL11121 | Breast | Unique | 0.029 | NA |
| **21:24895983** | C | T | BBL11121 | Breast | Unique | 0.033 | NA |
| **21:26729459** | G | A | BBL11121 | Brain,Liver,Colon,Ovary,Skin,Breast | Shared | 0.067 | 0.034 |
| **X:20373572** | G | A | BBL11121 | Liver | Unique | 0.048 | NA |
| **X:150909213** | C | T | BBL11121 | Breast | Unique | 0.027 | NA |
| **1:22225123** | G | A | BBLC1013 | Artery,Brain,Colon,Liver,Prostate,Skin | Shared | 0.048 | 0.028 |
| **1:64465989** | C | T | BBLC1013 | Artery,Brain,Colon,Liver,Prostate,Skin | Shared | 0.040 | 0.026 |
| **2:875456** | C | T | BBLC1013 | Liver | Unique | 0.069 | NA |
| **2:38366144** | A | C | BBLC1013 | Prostate | Unique | 0.039 | NA |
| **3:168112730** | T | C | BBLC1013 | Artery | Unique | 0.032 | NA |
| **4:67061519** | G | T | BBLC1013 | Artery,Brain,Colon,Liver,Prostate,Skin | Shared | 0.187 | 0.030 |
| **4:67129912** | C | A | BBLC1013 | Artery,Brain,Colon,Liver,Skin | Shared | 0.069 | 0.023 |
| **4:147210434** | T | A | BBLC1013 | Prostate | Unique | 0.021 | NA |
| **6:69672804** | G | A | BBLC1013 | Prostate | Unique | 0.042 | NA |
| **7:30959565** | G | A | BBLC1013 | Artery,Brain,Skin | Shared | 0.014 | 0.006 |
| **8:15332347** | C | T | BBLC1013 | Artery,Brain,Liver,Prostate,Skin | Shared | 0.167 | 0.025 |
| **12:118603305** | A | T | BBLC1013 | Artery,Brain,Liver,Prostate | Shared | 0.044 | 0.021 |
| **16:77628019** | C | T | BBLC1013 | Artery,Brain,Colon,Liver,Prostate,Skin | Shared | 0.076 | 0.027 |
| **17:78776823** | G | A | BBLC1013 | Artery,Brain,Colon,Liver,Prostate,Skin | Shared | 0.023 | 0.019 |
| **18:74589625** | A | G | BBLC1013 | Liver | Unique | 0.137 | NA |
| **22:24132092** | G | A | BBLC1013 | Artery,Brain,Prostate | Shared | 0.027 | 0.008 |
| **1:49035302** | G | T | BBLD1005 | Liver | Unique | 0.020 | NA |
| **1:244316714** | G | A | BBLD1005 | Artery,Brain,Colon,Liver,Skin | Shared | 0.051 | 0.013 |
| **2:105741925** | G | A | BBLD1005 | Liver | Unique | 0.021 | NA |
| **2:181549566** | A | G | BBLD1005 | Liver | Unique | 0.021 | NA |
| **4:56210847** | C | T | BBLD1005 | Artery,Colon,Liver,Skin | Shared | 0.035 | 0.013 |
| **4:82184505** | C | G | BBLD1005 | Liver | Unique | 0.014 | NA |
| **4:97078131** | C | A | BBLD1005 | Liver | Unique | 0.014 | NA |
| **4:97707271** | A | G | BBLD1005 | Liver | Unique | 0.022 | NA |
| **5:6810959** | G | T | BBLD1005 | Liver | Unique | 0.019 | NA |
| **5:18840767** | G | T | BBLD1005 | Liver | Unique | 0.022 | NA |
| **5:38097742** | C | T | BBLD1005 | Liver | Unique | 0.040 | NA |
| **5:59002752** | G | T | BBLD1005 | Artery,Brain,Colon,Liver,Skin | Shared | 0.145 | 0.037 |
| **5:108791612** | C | A | BBLD1005 | Liver | Unique | 0.027 | NA |
| **6:9117410** | C | G | BBLD1005 | Liver | Unique | 0.015 | NA |
| **6:113444094** | G | C | BBLD1005 | Liver | Unique | 0.028 | NA |
| **6:139842988** | C | A | BBLD1005 | Liver | Unique | 0.012 | NA |
| **6:143426551** | C | T | BBLD1005 | Brain,Liver,Skin | Shared | 0.016 | 0.012 |
| **7:101860696** | G | T | BBLD1005 | Liver | Unique | 0.023 | NA |
| **7:143002185** | C | T | BBLD1005 | Brain | Unique | 0.024 | NA |
| **8:13105971** | C | T | BBLD1005 | Liver | Unique | 0.027 | NA |
| **8:62581864** | T | G | BBLD1005 | Liver | Unique | 0.020 | NA |
| **8:99610896** | T | C | BBLD1005 | Liver | Unique | 0.036 | NA |
| **10:13562293** | C | T | BBLD1005 | Colon | Unique | 0.015 | NA |
| **10:36073362** | G | A | BBLD1005 | Liver | Unique | 0.015 | NA |
| **10:85183194** | T | A | BBLD1005 | Artery,Brain,Colon,Liver,Skin | Shared | 0.160 | 0.011 |
| **11:1391515** | C | T | BBLD1005 | Liver | Unique | 0.037 | NA |
| **11:76807693** | G | A | BBLD1005 | Artery,Brain,Colon,Liver,Skin | Shared | 0.031 | 0.012 |
| **11:106871001** | G | T | BBLD1005 | Liver | Unique | 0.024 | NA |
| **12:2324301** | G | C | BBLD1005 | Liver | Unique | 0.010 | NA |
| **12:26901510** | C | T | BBLD1005 | Liver | Unique | 0.065 | NA |
| **12:74691796** | G | C | BBLD1005 | Liver | Unique | 0.024 | NA |
| **12:87993265** | C | A | BBLD1005 | Liver | Unique | 0.025 | NA |
| **13:55948090** | G | A | BBLD1005 | Liver | Unique | 0.032 | NA |
| **14:21736571** | C | A | BBLD1005 | Liver | Unique | 0.021 | NA |
| **14:82146609** | G | C | BBLD1005 | Liver | Unique | 0.017 | NA |
| **14:96305274** | T | C | BBLD1005 | Artery,Brain,Colon,Liver,Skin | Shared | 0.152 | 0.039 |
| **15:25350138** | G | T | BBLD1005 | Liver | Unique | 0.042 | NA |
| **15:94590035** | G | T | BBLD1005 | Liver | Unique | 0.026 | NA |
| **16:3639261** | C | T | BBLD1005 | Brain,Colon,Liver | Shared | 0.032 | 0.022 |
| **16:18814565** | C | A | BBLD1005 | Liver | Unique | 0.021 | NA |
| **18:12104914** | G | C | BBLD1005 | Liver | Unique | 0.023 | NA |
| **18:38513947** | C | T | BBLD1005 | Liver | Unique | 0.022 | NA |
| **18:53608260** | T | A | BBLD1005 | Liver | Unique | 0.021 | NA |
| **20:9305925** | C | A | BBLD1005 | Liver | Unique | 0.038 | NA |
| **20:19541238** | G | A | BBLD1005 | Liver | Unique | 0.014 | NA |
| **21:46529741** | C | T | BBLD1005 | Liver | Unique | 0.028 | NA |
| **X:7442005** | G | A | BBLD1005 | Artery,Colon,Liver,Skin | Shared | 0.152 | 0.049 |
| **X:22516173** | G | T | BBLD1005 | Liver | Unique | 0.012 | NA |
| **X:25272450** | G | T | BBLD1005 | Liver | Unique | 0.047 | NA |
| **X:117758307** | G | A | BBLD1005 | Liver | Unique | 0.011 | NA |
| **X:125573982** | T | C | BBLD1005 | Liver | Unique | 0.032 | NA |
| **X:127329197** | T | C | BBLD1005 | Liver | Unique | 0.069 | NA |
| **X:132692389** | G | T | BBLD1005 | Liver | Unique | 0.020 | NA |
| **X:142880750** | A | T | BBLD1005 | Liver | Unique | 0.056 | NA |
| **1:5144704** | C | T | BBLD1010 | Colon,Prostate | Shared | 0.097 | 0.110 |
| **3:74385683** | C | T | BBLD1010 | Prostate | Unique | 0.076 | NA |
| **3:126380038** | G | A | BBLD1010 | Brain,Liver | Shared | 0.015 | 0.009 |
| **4:22712686** | G | A | BBLD1010 | Prostate | Unique | 0.087 | NA |
| **6:167609855** | C | T | BBLD1010 | Brain,Colon,Liver,Prostate,Skin | Shared | 0.246 | 0.008 |
| **7:49883392** | A | G | BBLD1010 | Skin | Unique | 0.017 | NA |
| **8:50367950** | G | A | BBLD1010 | Prostate | Unique | 0.098 | NA |
| **8:92360228** | C | T | BBLD1010 | Prostate | Unique | 0.090 | NA |
| **9:33270489** | G | A | BBLD1010 | Brain,Colon,Liver,Skin | Shared | 0.085 | 0.029 |
| **9:93652243** | C | A | BBLD1010 | Skin | Unique | 0.049 | NA |
| **9:117788988** | G | C | BBLD1010 | Brain,Colon,Liver,Prostate,Skin | Shared | 0.206 | 0.036 |
| **12:2027542** | C | T | BBLD1010 | Prostate | Unique | 0.196 | NA |
| **13:96860919** | A | G | BBLD1010 | Skin | Unique | 0.042 | NA |
| **15:89676236** | A | T | BBLD1010 | Brain,Colon,Liver,Prostate,Skin | Shared | 0.214 | 0.065 |
| **15:94441079** | C | A | BBLD1010 | Prostate | Unique | 0.064 | NA |
| **15:100647704** | C | A | BBLD1010 | Liver | Unique | 0.024 | NA |
| **16:66671706** | G | A | BBLD1010 | Prostate | Unique | 0.127 | NA |
| **17:62324984** | G | A | BBLD1010 | Colon | Unique | 0.102 | NA |
| **21:17967785** | G | A | BBLD1010 | Prostate | Unique | 0.117 | NA |
