# Supplementary material for: Distinctive types of postzygotic single-nucleotide mosaicisms in healthy individuals revealed by genome-wide profiling of multiple organs
Source: PLoS Genet. 2018 May 15;14(5):e1007395. doi: 10.1371/journal.pgen.1007395 (PMC5969758; doi:10.1371/journal.pgen.1007395)
Supplement: S3 Table — (DOC) [file pgen.1007395.s020.doc]

S3 Table. Chromatin state for the embryonic pSNMs that were globally present in all the sequenced organs of the individual and those only present in some but not all the sequenced organs.

|  | **% in transcribed chromatin status** | | |
| --- | --- | --- | --- |
|  | HepG2 | HMEC | K562 |
| **Embryonic pSNMs** | 36.7% | 36.7% | 33.3% |
| **-- Globally shared** | 37.0% | 37.0% | 22.2% |
| **-- Partially shared** | 36.4% | 36.4% | 42.4% |
| **Clonal expansion pSNMs** | 19.7% | 22.4% | 15.8% |
