# Supplementary material for: Distinctive types of postzygotic single-nucleotide mosaicisms in healthy individuals revealed by genome-wide profiling of multiple organs
Source: PLoS Genet. 2018 May 15;14(5):e1007395. doi: 10.1371/journal.pgen.1007395 (PMC5969758; doi:10.1371/journal.pgen.1007395)
Supplement: S4 Table — (DOC) [file pgen.1007395.s021.doc]

**S4 Table. Mosaic sites identified by cancer panel sequencing of BBLD1005’s liver samples.**

| **Position** | **Gene** | **Liver #2 (Panel)** | | | **Liver #8 (Panel)** | | | **Liver #9 (WGS)** | | |
| --- | --- | --- | --- | --- | --- | --- | --- | --- | --- | --- |
|  |  | Allele fraction | Lower 95% CI | Upper 95% CI | Allele fraction | Lower 95% CI | Upper 95% CI | Allele fraction | Lower 95% CI | Upper 95% CI |
| **4:55592222** | KIT | 0.0% | 0.0% | 0.1% | 1.4% | 1.0% | 2.0% | 0.0% | 0.0% | 2.7% |
| **9:121930001** | BRINP1 | 1.7% | 1.2% | 2.2% | 1.6% | 1.1% | 2.2% | 0.0% | 0.0% | 3.1% |
| **9:139398862** | NOTCH1 | 0.1% | 0.0% | 0.2% | 1.6% | 1.1% | 2.3% | 0.0% | 0.0% | 3.2% |
| **19:34713232** | LSM14A | 0.0% | 0.0% | 0.1% | 0.8% | 0.5% | 1.2% | 0.0% | 0.0% | 3.4% |
