# Supplementary material for: Distinctive types of postzygotic single-nucleotide mosaicisms in healthy individuals revealed by genome-wide profiling of multiple organs
Source: PLoS Genet. 2018 May 15;14(5):e1007395. doi: 10.1371/journal.pgen.1007395 (PMC5969758; doi:10.1371/journal.pgen.1007395)
Supplement: S5 Table — (DOC) [file pgen.1007395.s022.doc]

**S5 Table. Primers used for PGM Amplicon Sequencing of Mosaicism (PASM).**

| **ID** | **Position** | **Forward primer** | **Reverse primer** |
| --- | --- | --- | --- |
| **N9** | 8:17264659 | ATGGAGACTGAATGCCAATG | TGTTTCTTACTATACCATTCCATTG |
| **N8** | 7:49883392 | AGGGCTAGAGTCCTGCATGT | CCTGTGCTGCCAACCTTCAT |
| **N7** | 6:167609855 | GAACCTTCCCTGTCACCTCC | TTTTGTCGCCGTTCCTGAGA |
| **N6** | 5:155017653 | AAGTAGTTGGCAGAGTATGTCTGT | TTCCCACATTGAGCTTAACAGATT |
| **N5** | 4:22712686 | GCACGGTAGTTTTCTGTCACAT | TGTGTCATAGTCCATTGCTTGAG |
| **N4** | 3:136054013 | AGACCCTGTCTTCACTTACCG | AAGAAGTCATACCCTACACACTTC |
| **N39** | 18:48811808 | TGAAAATTTACAAAGAACGTCCTGA | TACCTGCACTCAGCATCAACG |
| **N38** | 6:167732359 | GGCCAGCTGGACCTATGAAG | TGTAGTGCGTAGATGATCTGATTGA |
| **N37** | 4:10096738 | CGCCTTCAGAACATGCCTTG | AAGTTCCACTTGCTGTGAAATCC |
| **N36** | 3:74385683 | TGTGCTGTGCTAAGGAAATATTGTT | ACTAGCTTAAGGCAACATGTTCTG |
| **N35** | 1:5144704 | TCCTTTCACATTCCCATTCACTCA | GCCAACATATGGATTCTAGGCT |
| **N34** | 13:71547014 | CATACTTAGCAACAACCATACAACA | TGATGAACTGAACGTTTGTCTTT |
| **N33** | X:63615302 | ACAGGAAAGACGCGTCAAAGA | AAGACTCGGCCAAACGTTGA |
| **N32** | X:33305280 | ATGTATGCAGATGACTCTGAGG | AAACATCCTACAATGCAAAGG |
| **N31** | X:17453925 | AATGTGAAAACCTGCCTC | TTTGATAGCAAGGATTTCAG |
| **N30** | 21:17967785 | TGGTGACTTTTTCAATTAGAGAGGA | TCACTAAGAGATAAGGTAGGGATCA |
| **N3** | 3:126380038 | TGAGCATGGATTTCCCCCTG | TCACACCTGGCAGTGTAGAAT |
| **N29** | 19:32523452 | GGAGAGGTCACCCAGTCAAGTA | CTCGAGCAATGTTAAAAATGAGGAA |
| **N28** | 17:74008696 | AGGCACCTTGGCAGAATCC | TGGGCCCTGCATGCCT |
| **N27** | 17:62324984 | AGCAGTGAGGAAAGAGAATGTC | TGTAATGAATTCTGTACATCTGGG |
| **N26** | 17:41441765 | ATTCTAATTTAATGGCTGTACGG | TTAGGTGATTAGGACTTTGC |
| **N25** | 16:66671706 | TTTCAGGAGAAGGAGGAGGC | CGGCATCATCAAGTGGAGCA |
| **N24** | 16:19808879 | TAAATAAGCGCCCGTGGTGT | ATCTGTCCCACCCTCAACCT |
| **N23** | 15:100647704 | ACGCACACTGAAATGTTGATG | ACCTCGCAAAACATCCACC |
| **N22** | 15:94441079 | ATCCTCTAAGGTTAGGGGTGAGA | TTAGGGTTAGTAAAGCCCTCTGC |
| **N21** | 15:89676236 | GAGAGATGGACTTTGCTTTAGTAGG | GGCAACACGGAGGGTACAAG |
| **N20** | 13:96860919 | CCTGCCTAATTTCCGAGGGTT | ACATGGGAAAAATCTGCCTTGTTA |
| **N2** | 2:121972323 | AGCGCCATCCGTGGGGCTTGG | TGCACAACGGTTCCAATTACTCC |
| **N19** | 12:2027542 | TGGGGTTGAACGTGATGCTT | CTAGGTGGGGCGACTCTTCT |
| **N18** | 11:29960713 | AGTAGCTTCTGGGAAAAACAGAAAG | AGAGAACTCTCTGTCGCTCTCTT |
| **N17** | 10:79980419 | TGTGAATGCAGTCTCAGAAGGC | AAGTGTGCTCATCTCTTCCCAAC |
| **N16** | 9:139221591 | CATTCCGGGGGAGCTTGG | CCCCTGGGGACTTCTGAGG |
| **N15** | 9:117788988 | TTCTGGATTGGCACTCAGTAGG | TGTACCCAGTTGCCGCTTTT |
| **N14** | 9:93652243 | GCAAGAGGGAAGTGCTACTCA | TTCCCACCCACCTTTCACTC |
| **N13** | 9:33270489 | TTAAGCGATGCATGACTG | TGTCTTTAAAAAGCCATCGG |
| **N12** | 8:121008897 | TTGACTATCTCATCTGGCCCTC | AAGACCTACTGTTTTCTCTTTGCAC |
| **N11** | 8:92360228 | GCCATGGTCATCAGACTCATTTC | GAATGCGGAGACAATCTCAGTG |
| **N10** | 8:50367950 | TGGAAGCCTACAGACTTTCAGT | CACGCCTAGGTCTCCACAAA |
| **N1** | 1:56946816 | AGAGTCATAGGGGCTTGGGT | GTTTGGATCAAGTTCAGACAGGC |
| **Z9** | 10:120489728 | ACACCAACAGATTAAGGTCTCCC | TGGATGGTGCCATAGATCAACT |
| **Z8** | 10:68687696 | TGGACCTGGAGCTCCTTACA | GCTTAAACGTCGGCTTTGGG |
| **Z78** | X:150909213 | TGAAAGCTCTGCCCCTTGAC | ACCAGTTGTAAAGGACGGGC |
| **Z76** | X:20373572 | CTCACCCTCGTCTTGTCTTGG | AATGATACCATTGGCCATTTTTGGT |
| **Z74** | 9:28943014 | GAGAGAGTGAGCAAATGGGGG | ATGACACTAGCATTCTGGGACC |
| **Z73** | 9:28463213 | CAGTGTATGTCTGCAAATCGTGG | TCTGCCATCCCACTCATTCAA |
| **Z72** | 8:131927641 | TATTTTTGCTCACCTCTGTCTAGC | CTAGCCTGACATCCACAAGCA |
| **Z71** | 8:119263974 | TCACCAGGTCTTTTTACGCC | GATTATGCTGAGCACACGGG |
| **Z70** | 8:109523811 | GTTTTCCATAAGCAACAGGGCA | TGTACTTAGTGATTCAGGAAGAGGA |
| **Z7** | 10:53107127 | CCTTGGAGTTGGGGTTAAGCA | CAGAGACATGGGTAAACAAGTTCAT |
| **Z69** | 8:100642472 | GGTGCAGATCACTAAGTCCCA | CTGGACATACCTAAACCAGGACA |
| **Z68** | 8:61243836 | GGATCCCCTATGTAGCTGGC | AAACAGCAAACATTTGGGTCTAA |
| **Z67** | 8:5881031 | TTGTCTAAGTATCCTGGGCATAGC | GTAGTGGAAGGCACTGAGCAC |
| **Z66** | 7:103982586 | AAGGGAAAAGCTAACTCCTCCTG | CCCTAAAGTTTCCCAAGAAATGGG |
| **Z65** | 7:79880023 | ACCAAGTGCGGCTGATTTTAC | AGTTGTCAACGGGCTTACAG |
| **Z64** | 7:68358382 | GGAGCCTGTCCAAAACCCAA | TACCTTGAGCGGCTGAACTG |
|  |  | ATCCGCACACCTTGGCTTC | TTTATCTCTTGCCTTCAGCCTCC |
| **Z63** | 7:1425148 | GATGTGGTCACCTGGCTGTT | GCTGAGTTGGGGGTTCTGAG |
| **Z62** | 6:170547487 | GAAGAAGTAACTCCTGGCGGA | TGTTTGCGATGGCCGAAAAG |
| **Z61** | 6:170452855 | CGGATTCTCCCGAAGTGTCT | GATCCTGTTTATGGCGCCTC |
| **Z60** | 6:143020735 | TGGGCACAAATCAACCTTGTC | CCATTCATTTAGACCCTTTCCTCA |
| **Z6** | 10:14807013 | TTAACTGATGCACAACTGCAAG | AGCACCGTTTATTTTCAAGTGAGG |
| **Z57** | 6:80434788 | ACATCATATGCTCTTCACTCCTC | GTGGTGGACTGCTCTGTATCA |
| **Z56** | 6:73468560 | ACTCTCAGCTGCCAACTAGG | ATGTGTAGTTTCAGTGGACTCTCT |
| **Z55** | 5:88341379 | CCTCATCAGATTCTTTAGGAGATGC | TAAGATCCACTCTCACAAAAGGC |
| **Z54** | 4:180446403 | CCCAATTTCAATAAACCTAGGAAGA | CCTACACAGCTAGGCATTTTTGT |
| **Z52** | 4:74106862 | AACCATAAAACCCCACACAGATG | ACCTTATTGGCCACTGAAATGTTA |
|  |  | ACTACAGGAGCATGCCACC | TCTCTTGGCTAGTGTGCCTC |
| **Z51** | 3:175619792 | TGCATTAAGAGGTAGGAGAAGCA | TCCTCTGTCCCTTGAAACAACT |
| **Z50** | 3:165204247 | CATTCAGGTGCTTTTATAGCTGGA | AGCAGGGATGACTCACTCTAT |
| **Z5** | 1:241471556 | TTGGATGCAAACAGCCTTACC | CCGTGTCAAAACACAAAATTGCC |
| **Z49** | 3:115320238 | GCAATTGGAATGAGGGAAATGC | TCAACTTAGTTTTCAGGGCCTTTT |
| **Z48** | 3:36701049 | TGATTTCATGTCCTGACTTCTCTG | TACTAACAGTAACAAAAAGGCAACG |
| **Z47** | 3:31252080 | TCTCCAGCCTCAATTTCCCA | TGTTGTTCCCAGGCATGGTT |
| **Z46** | 3:28336801 | CAGTGTTCACGCTGTGCTAC | AGCCAGTGATGTGATACCGA |
| **Z45** | 3:20903522 | AGATAAGGCAATTTCCCACAGC | ATCGCCTAACACTACCCCAAA |
| **Z44** | 22:46468495 | CTGCGAAGAGTTGCGGGA | AATGCCGAGTGGAGTTCTTG |
| **Z43** | 21:26729459 | CATTTGTTTGAGGCCAGCTGAA | AATGTGAGCCTGTTTAGAAGTCA |
| **Z42** | 21:24895983 | AGGACTTCAGAAGAAAGGAAGACT | ACTGTAGAAGCTTCAAAATGGGC |
| **Z41** | 20:61150548 | TATTGATCAGGGGCTGGTGG | AGGCGAGAAAGACGATGCTT |
| **Z40** | 20:15885200 | GGAAGGCCATAGTAGCGCA | AGGGGTCTTTCCATTAGCTTGC |
|  |  | TGCCCAGCCCAAATTTTCC | TTAGCCAGGATGGTCTCGATC |
| **Z4** | 1:234747291 | TTTTGCCCTATATGCCACCCA | CGACTAGAGAGAAACGTCAGCAT |
| **Z39** | 20:6174493 | GAAACCAGGCAAAGGTAGGGA | CTGTCGATACCAACAGGCGG |
|  |  | TTGCATACCAAACAGAGGCC | ATGCCTGTAGTCCCAGCTAC |
| **Z38** | 2:236800392 | CAGTTCAGGTGGATCGAGGG | ATGCACGTCTGCCAGGTATG |
| **Z37** | 2:227430472 | GCTGATGCTACTACCTAAGAGGC | TCTTTGTTCTTTATCCACAGAGGAA |
| **Z36** | 2:226710617 | ACTGCTAAAGAAAGTAAGGCATGA | TTCTGATTTTCTTCTACTAGCTCCA |
| **Z35** | 2:126487527 | AACTCCAAAGTTACTTCAAGGCAA | ACTGACCAGAGACTTAGAAAGCC |
| **Z34** | 2:47722338 | CTGGGAGGGGCTTCAAACAA | GGCTATAGCAGCATACAAGCTC |
| **Z33** | 2:46135258 | CAAAACGCCTTGTCCAGAATGA | TCTTCCCAGTGCCTAGGAGTTA |
| **Z32** | 2:3913156 | GGCATGGATTGTCGAAGGGA | TACCCTCGCAAAGGAAACCT |
| **Z31** | 2:3260480 | TTGTCACCTTGGTGTCCCTG | CGGCATGTGGTGTGTCATTC |
| **Z30** | 19:48525633 | GGAATTTCCGCGTTGGTCCC | TGGGGAGTTGTTCCAGTCTTC |
| **Z3** | 1:99953413 | ATTGCTCAAACTTATGTGGGCTA | ACTGATAGAAGAACGGAAGCCCT |
| **Z29** | 18:75697941 | GCTTTTATTGGCTGGGAGGATG | GAGGGGGTTCTCGGCTGAT |
| **Z27** | 17:52228988 | AGCTGTCCTAAAATGGAGAGGC | GCTTGTGTCATCTATGTAGGAGC |
| **Z26** | 17:7077804 | TTACCATTGCCCTGGAGCG | TGTTTTGTGTGTTGTCCGTGTG |
| **Z25** | 17:2084756 | GTAGTCAGGCCAGGTTTGCT | GGGCTTCACATTCTGGGCTA |
| **Z23** | 14:74665394 | ACCAGCAACCCAGAGAAGAC | CTCTGGCTGGAATCACAAAGC |
| **Z22** | 14:66089975 | TGCAGGAGCAAACCGAAGT | TGAGACCAGGTCAAGGTTTGG |
|  |  | TCGGTCTTGAACTCCTGTGC | TGTGATCCACCCTCCTCGG |
| **Z21** | 14:50549268 | CACAGTAAATCTGTGAGGTTGCT | AGCAGGTACTTTAATAATCTAGGCT |
| **Z20** | 13:104700276 | CCTTCTGATTATCTAGGCCATGCT | TCCACAAACATTCTTCATCATTGT |
| **Z2** | 1:50640673 | CACTCGTAAGCATGATTCCTCC | GTGTCACCTCATCAAACCTAGC |
| **Z19** | 13:82873560 | ACGCTTGCTTTTATGTCTGTGTCA | GTTTTCTTCTGTACCCCCACCTAT |
| **Z18** | 13:79395067 | CAAGCTAATGAGGCATGGAGC | AAAAACATAGGTCCCAGCGAGG |
| **Z17** | 13:72778058 | ATTACCCTTCTCCAATTCCATTTAC | TGTCTTTGATATCTTGGCACTCT |
| **Z16** | 13:23910741 | GGACTGCTCCTAGTTTTACTGCT | GAGGCGGCACCATACCTTTA |
| **Z15** | 13:23780127 | ACCTGTTGCTAATTGGTAGCG | TGTTAAGAGGGCAGATCTTGTG |
| **Z14** | 12:130128978 | TGCCCCCTTTTCTAGTGGATG | TGTAACGGCACTGTAAAATGGT |
| **Z13** | 12:79409138 | GTTTCAAAGCTAAACTTGCACTCT | CGTTTAAGCAAATAGGGAGCGA |
| **Z12** | 12:53614017 | TACATCGCGACCCGGCTT | GCGGAGCGAGTGTACGTATG |
| **Z11** | 11:132113942 | CCAGTAGTCCACAAGAGGGC | CTGGCCATCCCTCCCTTCTA |
| **Z10** | 11:131717828 | GCCTCAGAGCTCAGCAGTAG | ACATGATACTCAGCAAACGTCA |
| **V9** | 4:41634722 | CCTCGAGGCCTTGAATCCTG | CACTGCTCCTCACCTTCCTC |
| **V5** | 2:135722990 | AGCTATTGTGATTGAAAACTAGGC | GGCCAATTTAACCAAAAGAATGCC |
| **V39** | 16:13019024 | AGAATGAGTGACTATTGGTGCGA | TGCAAATATAAGACATGCTGGGG |
| **V35** | 6:164101476 | GTGTGAGACATAGATAGCAGCGA | GTTGGTTGGTTGGCATAACCC |
| **V33** | 5:125465949 | ACCCATAACTGCACCCTACG | GAATTGAGCAGATGTTTTCATCCA |
| **V31** | 5:64603697 | CATATACAGCAAGGCTCCACTCT | AACATCATGGAAGTCCTGTGGA |
| **V30** | 2:228568980 | TACATAGCACTCAAGGAAGATGCT | TGACAACCATACCACCTCCA |
| **V3** | 2:53378193 | TACAGAACCAGTCAAGCGCC | TCCTTCATGACTTCCTTTTTAGAGA |
| **V25** | 1:114973790 | AACTGGGCACTTAACCGAGT | AGAACCCATGTTTGAAGTGGC |
| **V23** | 20:40919410 | TTGGTAGAATAGGCAGGCCAC | GCCTAACCCAAGTCTTTCTGGAT |
| **V21** | 17:68638247 | CATGTGGGATCTCATTGGCT | ACACACAAGCTCCTTGTCATTG |
| **V2** | 1:224876814 | TGACACAGAAGATCTCTGACCT | GTGTGCTTAGTGATGTCATAAAGGG |
| **V19** | 14:37786847 | GAGATTTTAATGGGTGAGGGACC | AACCACTTCTGAATCTGGCTTACT |
| **V18** | 11:134115864 | GCCTCTCTGGGGAACATGAG | ACAGGCTTCTGTTCCAGGAC |
| **V17** | 11:95826599 | GTGTATGGGATGGCAGAGTGTTA | ATTGTGCTTCATTTGTGCTCTCTC |
| **V16** | 11:40616679 | CACACACACAAACACGCCAT | TCTTTCCAGCATGTAAGTCCCT |
| **V14** | 6:93892544 | TCTGGGAAGCCACCTACATA | CAGGCAAATTAAAGTCATACCTAGC |
| **V10** | 4:181048637 | ACCGTGCATTCAAAAGTGCATTC | CAATCACAATGACAGTGGCCTTT |
| **Q9** | 3:168112730 | AGCTCTTAGGAATTATCTCCCAACC | GTCCACTCCAAGATTTGGGC |
| **Q7** | 3:61685928 | GAGGTTTCATGCCCAAAGTCTC | GGGAATTCACTGAGCCCAATTT |
| **Q6** | 2:105437531 | ATGTCCATCAGGGTCTATGCC | AAACTGGCTTCTCAGGGACTG |
| **Q5** | 2:38366144 | CCAGGGGTTCACCCAAGTTT | TAAATGCGCAGCTGTCCTTG |
| **Q4** | 2:875456 | ACACGATGCATGTTTCTGCC | ACCCAAAACAGGGATTACGCA |
| **Q35** | 22:24132092 | TCCTGAGACCCAGGCTGTAA | TCTTGGGACCCAGGTTTCCT |
| **Q34** | 20:40404525 | CTGGGGGAACATTCAGACCT | TGATGTGTTTTGCACATGGCTT |
| **Q33** | 19:14876348 | TCCTGTCCTTACCTGGATGG | ACACGGAATCCCGAATAACC |
| **Q32** | 18:74589625 | AGAAAATTAGTTGTTGATACAGAATATC | TTTACCATTCCATTCAAAATGACAG |
| **Q31** | 17:78776823 | TATGGAGGCACACGTCACTG | GTGATGAGCACCGGAAAAGC |
| **Q30** | 16:77628019 | TGGGGAAGGTATCTGGTCTACTT | ATCCCCAGTTGTAGCAGGAAC |
| **Q3** | 1:160279053 | CAGGATTCTGGGAGTCAGCAT | GTTGATGAGTCCTCAGGGTGG |
| **Q29** | 16:1408022 | AGAGAGTTTGAAACATCAGTCTGG | GTGCTGACGGTTGCAGG |
| **Q28** | 14:48946443 | AATTGTGCTAATATGGAGGTGG | TATAATCACTTAACTAAAGACAGTAAG |
| **Q27** | 13:111117672 | CACAAAAGCCTGTGCCTTCG | GGAGTACCCTTCGTTCCAGG |
| **Q26** | 12:118603305 | TGGCACCAAAGGATGGAGAG | AACTGACTCACACCCTTAGCA |
| **Q25** | 10:14348323 | TCTCTGTTCACACAACTGTTAGATG | ATGAGCAGAGGCCTGGTAGA |
| **Q24** | 9:111591797 | ACCTGTAGGGTCATGTTGG | TTTTGGTGTATCTATATTATAATTTGCAG |
| **Q23** | 8:15332347 | TGGGGGATTTGGATCACACT | AGCAATTGGCCATCGTCAAAA |
| **Q22** | 7:157206990 | CAGTGGCAGCAGTTGCTTTA | CCTCAAGCTACGGTTACGGA |
| **Q21** | 7:51076291 | TTCTTCGCGAGGCTTCTTGT | CCTGTCCCGAGCATTGTCTT |
| **Q20** | 7:30959565 | CAACAGGCCTTTGAGTGCCC | CATCCCTCACAGATGTGACCA |
| **Q2** | 1:64465989 | TACGTGGGCCATACAAAGGG | TGGACCCAAAGTTAACACTCCC |
| **Q19** | 7:18893103 | GACCCTGGGGAGGGTTAAATC | GTTGAAAAGATTCTGCTGCCCT |
| **Q18** | 7:895629 | AGACCTCCATGGAGTGAGAC | TATGAGAAATAGTCTACTGAAACACATAAG |
| **Q17** | 6:69672804 | GAGCTTAATGTTTACCAAGACACT | CTTACTGGAAGCTGGTGGCA |
| **Q16** | 5:114761072 | TCATTTCCTGTGCCTTCTAGGAC | TCCAGTTTAGAGAGGCCGTG |
| **Q15** | 5:39255635 | ACTTGCCCAGTCTATGTGAC | TGGTAAAGTTTGTCCTCTGCGT |
| **Q14** | 4:147210434 | GAGGAAACTTCCAAGTGAGGC | TGGTAGGCTGATAACTAGAGTAAC |
| **Q13** | 4:67129912 | ACTTGGCTGTACAGGGGTC | ACGGTGAAACCCCATCTTTAC |
| **Q12** | 4:67061519 | ACAATTTTTGGAGATACTGGGGA | ATCATGGGCATAATGCGGCT |
| **Q11** | 4:62668367 | ACGGGCATCTTCAAACCTGTAT | TTTGGGGAAACAAGCAAAGCA |
| **Q10** | 4:13762161 | ATGTGACATCCATTGTGTGGG | TTTTATGCAATATTCAGTCTGGGG |
| **Q1** | 1:22225123 | TGTGGACTTCTAGGCTGTGC | CAATGAGAGGGGGATTGGCA |
| **P9** | 3:182243274 | TTGGCTTTATTGCTTGCCCTC | GCACCACATTTTCCGAACCC |
| **P8** | 3:132118085 | ACTGCTTGAGTGCACCAGAT | GTGGTTGAACCCTCTGAGCA |
| **P73** | X:142880750 | TTTAAGCTGCCAATGCTGCC | CCAGTGACTCAGAACAACCCA |
| **P72** | X:132692389 | GGAGAAATGTGAAGCCCCCT | GCCTCAGGTAATGGCGACAA |
| **P71** | X:127329197 | AGGCAATATTTCACTTTCATGGC | GCTCACATGGTTGCCTTTCAA |
| **P70** | X:125573982 | TCCAGGACTCAACCCCAGAA | TGTTTGTGTGTTTCAGGTATCAACC |
| **P7** | 2:181549566 | TAACAGTGAGATTGCAATAATGG | AGTTTAGCTTGGATAAATTTCTCCC |
| **P69** | X:117758307 | CATCTGCTTTATAGGCCAACTTCTA | ATTAGGCATGGCTGCACAAGA |
| **P68** | X:106872021 | TTGAGTCTGTGGCCGACTTC | CCGCACGGTAAGAATGAACG |
| **P67** | X:25272450 | CTGAATCCTCAGAAGGTCTCCA | GAAGTGGGACAAGACTCACCA |
| **P66** | X:22516173 | TCCTGAGCCACAGGGATATT | GCATATGACTTCCCACCAGC |
| **P65** | X:7442005 | TGTTTTAATACCAGTGTAAGTTGGC | AGGGCAATCTGAAGTTTAAGAG |
| **P64** | 21:46529741 | ACTGGAGGCTCCGTGTAGAT | TTGCAGCTGAAGCGTGACTA |
| **P63** | 20:19541238 | TCTTAAGTTGTCCATTGTCAGAAGT | TATTAAGTGGCTTGGATGGAGAA |
| **P62** | 20:9305925 | ACTTATCAGTTTGCGGCGGT | GGATGCTCAGATTCTGGGAGG |
| **P6** | 2:146355452 | GAGTGAAAAATAGCATACTGGATGT | CCTTTCCTAAGACTAAAACACAGGT |
| **P59** | 18:53608260 | TGACATACATATCTCCTGTTGGTCT | AAGAAGAGATGGGTTTTGGGGG |
| **P58** | 18:38513947 | CTGTGTCCTTCAGTACCTACCA | GGGAAGAGCGTGGCATCATT |
| **P57** | 18:27873588 | CTGAAATATGACTTGAAGGCAAAGA | GCAAAATTAAAATCTGCCCATAAGT |
| **P56** | 18:12104914 | TGCACCTGATTGTCAGGAAAAG | TGGATCACTAAACTGAGGCACAT |
| **P54** | 16:18814565 | GGAACTGTGTTGAGGCACTTG | TCTGCAGCTATGTTCTCTCACA |
| **P53** | 16:3639261 | AAAGCTCGGCCTGCTATTCC | AGGCGGTTCCTGAAACACTC |
| **P52** | 15:94590035 | GTGGTGAATGTGCGATTGGC | TTCGGAGCAAAGAAACCCCA |
| **P51** | 15:25350138 | CCCACTCACATAGCTTACAGCA | TCCACAAATAGAAAGTACCCTTCA |
| **P50** | 14:96305274 | GTCCGTTGGAGCACTCTGAA | CAGGCTCATGGAGGCTTTGT |
| **P5** | 2:105741925 | TAAGAGGCCACCCTGGGTTC | TGATCCTGCCTCCTCCTCAT |
| **P49** | 14:82146609 | TTGGTGACAAATTGCTGCG | AGGCACTTCCTCAGATTCCC |
| **P48** | 14:21736571 | TCAAATTTCAGCACCCAGCA | CAGACGGAAACTGAGGCAGA |
| **P47** | 13:105360675 | GACTGAACCATCACCTGACTGA | AGGGCTGATTGTTGGGGTTC |
| **P46** | 13:72148004 | TCTAGGGAAATAAGCCGAGGT | TGCATATTTAATTTTTGCTTTGGCA |
| **P45** | 13:55948090 | GCCCTAGCATCCTGAACAAC | TGAAACAAACCCTGTGGGAT |
| **P44** | 12:87993265 | AAATTGGAGGGGTGGGTGAG | GCAGCTTGTTCCTGCTCTGA |
| **P43** | 12:74691796 | TAGGGAAAGGGAGTTAGGGCT | TTGTAGCTGTGCCATCGTGA |
| **P42** | 12:26901510 | TGAGGTTACAGGTGTGAGCC | AATCCCACCTACTGTTTCACAG |
|  |  | GTGCTACTCCAGTGTCCGAA | GTCCGAGTGGTTGTTGCTTT |
| **P41** | 12:24215125 | AAGTTGCATCAACTAATTGCCA | GTCCAGTGAATTTCTTTTCGGCA |
| **P40** | 12:2324301 | CAGTGTCTTCTCCCCACGTC | CACCACCTGATCCACTGACC |
| **P4** | 1:244316714 | CCAGCTCCTACTCATCAGGC | GCTGCAACCATGATGAGACAAA |
| **P39** | 11:106871001 | CCCATCCATTCTCCTTCAGCA | ATGCCTCCAAAGGCATTGTT |
| **P38** | 11:76807693 | GTTACTTTCCCTCCCCAGCC | TTCGACTGGGACTCTGGTCA |
| **P37** | 11:1391515 | GGTGAGGCTCATCCTGTCTG | CCATGCCTGGGACTCCTGT |
| **P36** | 10:85183194 | TCTGTCTCCTTCCCTGATCCT | TGCCTCATTCGTGTGTTGTT |
| **P35** | 10:80628845 | TCTGCCTTGTTCCTGGAGGT | GGATTCCTCTGCGCTTGTCT |
| **P34** | 10:36073362 | CCTGAGACCATTACAGCCAGG | TCAGGGTTTGCTCATTGTGGA |
| **P33** | 10:13562293 | AAAGAGAGAGGAGAGCGGGG | TGTAACCGCCTTCCCTTGAG |
| **P32** | 9:38065989 | GATCACAACACTCTCGCAGC | ACGGGTCTTCTCTGTGTCCT |
| **P31** | 9:21874057 | AGTGTTCATTTTACCTCAATCAGCA | TCCTATTACTTACTGCTTTAGCCTT |
| **P30** | 8:99610896 | ACACCGAGCATACAGTCCAA | AACAAACACAAGGATAATCATGTCA |
| **P3** | 1:204943390 | TTGAGGGCAGAGGACGTAGT | TTCCCAGGTCTTGCTGAACA |
| **P29** | 8:62581864 | AAGTCCCCAAAGCCATCTGA | ACACTGTTTTTGGCAGAGGTT |
| **P28** | 8:13105971 | CTTCTGCACCTGTTGGTCCT | CTTCTGAAGGAAGCACCCCA |
| **P27** | 7:143002185 | ATAGTCCGTCTCCCCTTCCC | GAAAAGGAGCTGGCCTGGAG |
| **P26** | 7:101860696 | ATCTGTGTCACCCAGTGTCC | CCCTCTGGGGATGGTTTTCG |
| **P25** | 6:143426551 | CCATGCCACAAGAGCCAGAT | GACTGTGCTTGTGGACACATT |
| **P24** | 6:139842988 | AAACCATCACTAACCTGGCCC | CCATCTCCATAGCCTAATCCCC |
| **P23** | 6:127327908 | AGGGAGGCCAAGCAACAAAG | CCCATTAAAACCCCCTACTGTGA |
| **P22** | 6:113444094 | TGTTTTAAGGCAGGAGACACTTG | CTGCACCTCTTTTGGTCACT |
| **P21** | 6:9117410 | AGAGTCAAAAGGCAACCTGC | GGAGTACTGATGGGGGTGGA |
| **P20** | 5:108791612 | AGACTTTCTTCATCCACAAATGGC | TTAGCTGCTCAGGGCCAGTC |
| **P2** | 1:69794094 | TGCTCTCCTCAACTCCTGTC | GCAATCTCATTTACAACAGCTAC |
| **P19** | 5:59002752 | CCGAGCTCCTTCTCCTTATGC | TGGTTAGTGATGTCTCACTTCTGT |
| **P18** | 5:38097742 | TTGCCTACTCAGGCATCAAA | GCTTGGGTATCACTCTCCCC |
| **P17** | 5:18840767 | TTTCTGCTGAGTGGGAAGCC | TGGATAGAGGCAAGTTGTGCT |
| **P16** | 5:6810959 | GGTAGATTCTGTTGTGGCCT | TGGGAAGGGACGTGATCCT |
| **P15** | 4:97707271 | ATGATCCAGAGAAGTGAACACTGA | GGCAAGAGGGTTCTTTGGTT |
| **P14** | 4:97078131 | AGCATCCAGGACAGTGAGAC | AAGAGAGACTTTATTGCTATTGCTATC |
| **P13** | 4:82184505 | CAGAGGTTAAAGCCCGTCTGA | TGGAGCCTTTTGCTTGCTCT |
| **P12** | 4:76089625 | ACTCAATTGAGTAAAGGCCAGTATC | TTAACAAATTAACCAGGTGTGATGG |
|  |  | GCCCAACATGGACTTGCTTAC | GCTGCGATTTTTATCACAGGGC |
| **P11** | 4:56210847 | AAGATCGTGCCATTGCAC | TCTCCTGACCTCGTGATCC |
|  |  | ATCTACAGGGGGCTTCTCTGA | AGGCTTTACCCGTCCTTTGA |
| **P10** | 4:966497 | TAAGGATAGCCCCTCCCACC | CCCTAGCCCTTCTCATGCTG |
| **P1** | 1:49035302 | AGTTACACAGGCTGTTTCTGGT | TGGTCCCATTTGGTGATGGC |
| **L71** | 20:22982519 | CCCCACTTTCCTGACGACG | ACTCCCCCTTAGTACAGAGACTG |
| **L65** | 16:35039389 | AGACCCTTCAGTGAGACTCAGG | TTGGGTGGAGGGAATAAACTCCT |
| **L54** | 11:39703064 | AGTATTCTGCCTAGAGAACGCA | CTTGGGTGAAACCAGGTGCT |
| **L51** | 10:15186032 | ACTTGAGGGGATCTGGAGACA | GGGAAGCTCTGCCTCTCATC |
| **L48** | 7:128001041 | CAATCTGGCAGAAGCACCCT | GAACCCATGCAGGGAGGAG |
| **L36** | 5:157642290 | TGAAGACCTTCCTGTCCATGC | GCACTATTCCAGGTGTTAACTATGC |
| **L27** | 4:154773423 | GGCATAAGCAAGGTCCGTCT | GTCTCTCAAGTCTGGGCTCG |
| **L2** | 1:26769815 | GACGGGCTTATGGATCTGGC | ACCGGGGAGCAATGTCTGTA |
|  |  | AGAACAAGTACAGCCAGCCT | GCCCCTCTCTGGCTTTATTCC |
| **L19** | 3:12394900 | GTGGTAAGGGATGGTTATGTGA | TGGCCTCGGCTAATGATGA |
| **L11** | 2:121071503 | AATCCCAGCCAACTCTTCCTC | GACAAGATGGTCACCACGAC |
